# Small-Molecule Inhibitors of the m7G-RNA Writer METTL1

**DOI:** 10.1021/acsbiomedchemau.3c00030

**Published:** 2023-12-12

**Authors:** Francesco Nai, Maria Paula Flores Espinoza, Annalisa Invernizzi, Pablo Andrés Vargas-Rosales, Olga Bobileva, Marcin Herok, Amedeo Caflisch

**Affiliations:** † Department of Biochemistry, University of Zurich, Winterthurerstrasse 190, CH-8057 Zurich, Switzerland; ‡ Latvian Institute of Organic Synthesis, Aizkraukles 21, Riga LV-1006, Latvia

**Keywords:** epitranscriptomics, m7G writer, X-ray crystallography, enzymatic assay, docking, MD simulation, drug discovery

## Abstract

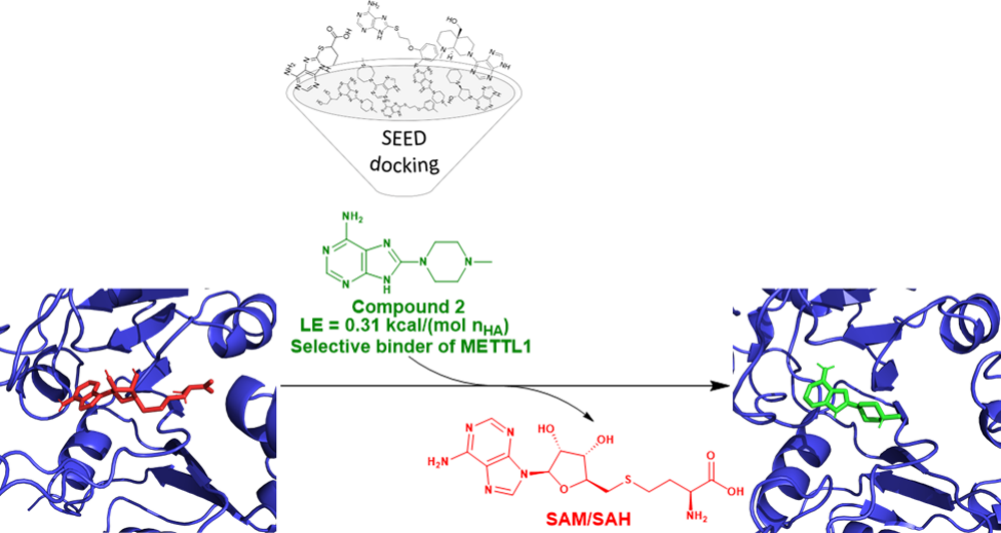

We discovered the first inhibitors of the m7G-RNA writer METTL1 by high-throughput docking and an enzymatic assay based on luminescence. Eleven compounds, which belong to three different chemotypes, show inhibitory activity in the range 40–300 μM. Two adenine derivatives identified by docking have very favorable ligand efficiency of 0.34 and 0.31 kcal/mol per non-hydrogen atom, respectively. Molecular dynamics simulations provide evidence that the inhibitors compete with the binding of the cosubstrate *S*-adenosyl methionine to METTL1. We also present a soakable crystal form that was used to determine the structure of the complex of METTL1 with sinefungin at a resolution of 1.85 Å.

## Introduction

The epitranscriptome is defined as the ensemble of chemical modifications introduced on RNA after transcription.^1^ Epitranscriptomic modifications are deposited by writer proteins and, with some exceptions, can either be recognized by reader proteins, leading to a biological effect, or erased by eraser proteins.^2,3^ Several epitranscriptomic modifications have been characterized: from the widespread and well-studied N^6^-methyladenosine (m^6^A) modification of RNA to the rarer and less known N7-methylguanosine (m7G).^4^

Methyltransferase Like 1 (METTL1) is an *S*-adenosyl methionine (SAM) dependent methyltransferase that forms a complex with WD Repeat Domain 4 (WDR4). The METTL1-WDR4 heterodimeric complex is the author of the m7G modification of RNA. METTL1 serves as the active core of the complex as it catalyzes the methyl transfer from the methyl donor SAM to a guanosine acceptor substrate which results in m7G-RNA and the demethylated byproduct *S*-adenosyl-l-homocysteine (SAH). WDR4 has scaffolding and ribonucleotide binding roles and it is thus essential for the activity of the complex.^5,6^

The m7G modification is deposited on transfer-RNA (tRNA), messenger-RNA (mRNA), and micro-RNA (miRNA), exerting variegated effects.^7−10^ Currently, no readers of internal m7G have been identified and, where more extensively characterized, the modification seems to directly exert its effect in a reader-independent manner.^5,6,9^

The m7G at position 46 is observed in most tRNAs in a large variety of species. The modification localizes in the variable loop of the tRNAs exerting a stabilizing effect through the interaction with cytosine 13 and guanosine 22 in the D-loop.^11−13^ This interaction is essential for tRNA stability, and an m7G writer is therefore present in organisms from every domain.^6,14−16^ The m7G deposition also affects mRNA and miRNA. The study of METTL1-WDR4-mediated m7G deposition on pri-let-7e pri-miRNA led to the discovery of a new paradigm in the field of epitranscriptomics, establishing the role of m7G as a “molecular handle” which destabilizes the G-quadruplex RNA secondary structure. The m7G-mediated destabilization of this non-Watson–Crick base pairing structure leads to substantially improved DROSHA/DGCR8-mediated pri-let-7e maturation.^9^

The METTL1-WDR4 complex is involved in developmental pathologies such as microcephalic primordial dwarfism^17^ and Galloway-Mowat syndrome^18^ as well as tumorigenesis. Overexpression of METTL1-WDR4 causes malignization of mouse embryonic fibroblasts while METTL1 knock-down or knock-out suppresses tumor growth in several xenograft tumor models.^19^ The increased expression of METTL1 was recently correlated with unfavorable prognosis in lung and hepatocellular carcinomas acting via the AKT/mTORC1 and PTEN pathways, respectively.^20,21^ Importantly, several publications have linked METTL1-WDR4 dysregulation to carcinogenesis^22^ suggesting that targeting the complex hold great promise for the development of chemical probes and chemotherapeutic drugs. The number of epitranscriptomic targets with an active program of drug discovery is very small. Inhibitors were disclosed for the m^6^A writer METTL3-METTL14^23−25^ and even more recent is the discovery of fragment binders of the m^6^A readers YTHDC1^26^ and YTHDF2.^27^ The innovative nature of the field and the role of METTL1 in oncogenesis make it a favorable target.

Here we set out to identify small-molecule inhibitors of METTL1 by high-throughput fragment docking and a medium-throughput luminescence-based biochemical assay.

## Results

We decided to focus on the SAM cosubstrate binding site which features good druggability.^28^ We first report the *in silico* screening (high-throughput docking) of a library of 4896 adenine derivatives. Nine compounds predicted by docking were tested experimentally (Table S1). We also carried out *in vitro* screening of 69 adenosine mimics (Table S2) and 26 adenosine derivatives (Table S3) by a luminescence-based enzymatic assay. These screening campaigns resulted in two, three, and six inhibitors with IC_50_ < 300 μM from each respective class (Table 1, Figure S1). Thus, the hit rates are 22% (*in silico* screening), 4%, and 23%, respectively. The binding modes of four compounds with favorable ligand efficiency (and belonging to the three chemotypes) were characterized by molecular dynamics (MD) simulations.

**Table 1:**
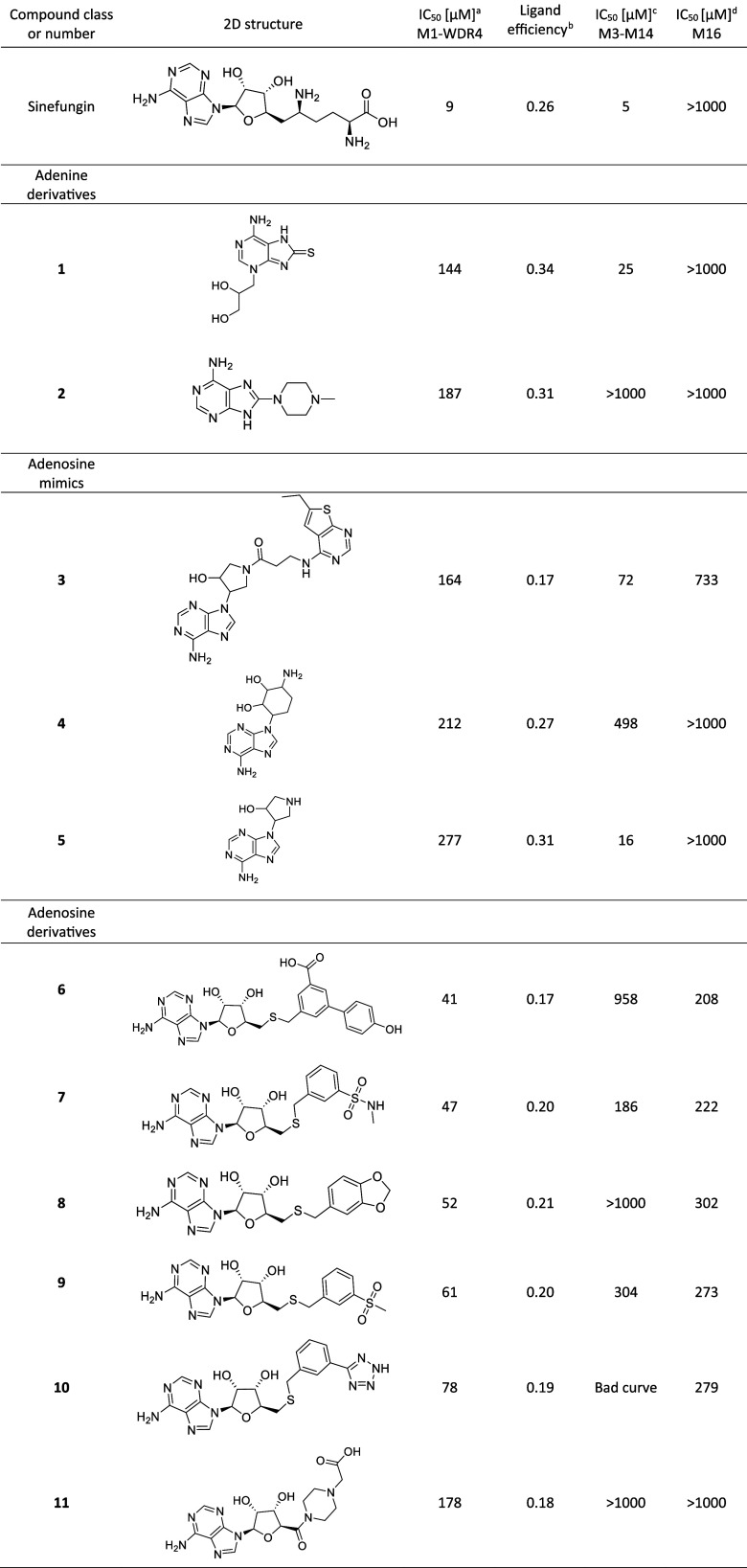
2D Structures and Affinity of Sinefungin and the 11 Small Molecule Ligands of the METTL1-WDR4 Cosubstrate Binding Pocket

a The IC_50_ value is measured using the luminescence-based METTL1-WDR4 enzymatic assay. The signal decreases when the small molecule competes with the binding of the cosubstrate SAM. The reported values come from the averaged curve of ≥2 biological replicates, each replicate is the average of two technical replicates (Figure S1).

b Ligand efficiency calculated according to

c The IC_50_ value is measured using a homogeneous time-resolved fluorescence-based METTL3–14 assay.^45^ The reported values come from the averaged curve of ≥2 biological replicates, each replicate is the average of two technical replicates (Figure S2).

d The IC_50_ value is measured using the luminescence-based METTL16 enzymatic assay. The reported values come from the averaged curve of two biological replicates, each replicate is the average of two technical replicates (Figure S3).

### High-Throughput In Silico Screening of a Library of Adenine Derivatives

We used high-throughput docking as a primary screening methodology for a library of 4896 adenine derivatives with number of heavy atom (n_HA_) in the range 15–23. For each compound up to 20 conformers were generated automatically and the docking was carried out with the program SEED.^29,30^ The structure of METTL1 (PDB code: 7OGJ) was kept rigid during the docking and the evaluation of binding energy. SEED calculates the binding energy by a force field with implicit treatment of the electrostatic effects of the solvent.^31,32^ Pose generation and energy evaluation required about 1 s per fragment. The SEED program package is available as open-source code from GitLab (https://gitlab.com/CaflischLab).

The docking poses were ranked on the basis of the binding energy calculated by SEED. The top 25 molecules were selected; nine of these were available for purchase and were tested with the enzymatic assay described below (Table S1). Compounds **1** and **2** resulted in a residual signal at 1 mM ≤ 50% and an IC_50_ of 144 μM and 187 μM, respectively (Table 1, Figure S1). Compound **2** shows promising selectivity against the m6A-RNA methyltransferases^3^ METTL3–14 and METTL16 (Table 1, Figure S2 and S3). The docking predictions of inhibitors **1** and **2** were corroborated by multiple MD simulations (see below).

### METTL1-WDR4 Enzymatic Assay and Screening of Small Libraries

In parallel to the docking campaign, we established and optimized an enzymatic assay useful for the screening and characterization of binders of the METTL1-WDR4 cosubstrate binding pocket. The assay exploits the full-length METTL1-WDR4 complex and a shortened version of the previously cited pri-let-7e with sequence: 5′-GGGCUGAGGUAGGAGG-3′ (from now on addressed as rG4-let-7e, in accordance with ref.^9^). This sequence has been reported to have sufficient length for the G-quadruplex formation.^33^ Several pieces of evidence corroborate the already reported complex formation between METTL1-WDR4 (ref.^9^) and pri-let-7e. The rG4-let-7e marked with the fluorescence energy transfer (FRET) acceptor XL665 can bind EU^3+^-marked GST-METTL1 leading to FRET and therefore implying close proximity and complex formation (Figure S4A)].^34^ The same oligonucleotide is also able to bind and stabilize both METTL1 and METTL1-WDR4 in thermal shift assay and a similar stabilization can be observed for both the proteins in the presence of a 120 bp version pri-let-7e (sequence: 5′-CUGUCCACCUGCCGCGCCCCCCGGGCUGAGGUAGGAGGUUGUAUAGUUGAGGAGGACACCCAAGGAGAUCACUAUACGGCCUCCUAGCUUUCCCCAGGCUGCGCCCUGCACGGGACGGGG-3′) (Figure S4C). METTL1-WDR4 binds the 120 bp version of pri-let-7e to create a stable complex, as clearly shown by analytical size exclusion chromatography (Figure S4D, E).

In the enzymatic assay presented in this study, the rG4-let-7e oligonucleotide binds to the METTL1-WDR4 complex, acting as a methyl transfer acceptor leading to SAM consumption and SAH production which in turn leads to luminescence emission. This can only be observed when both METTL1-WDR4 and rG4-let-7e are added to the reaction (Figure S4B). When a molecule able to compete with SAM is introduced, the enzymatic reaction is inhibited, leading to a reduction of luminescence emission. The assay was initially used to quantify the IC_50_ of sinefungin (9 μM, Figure S1) and, subsequently, to measure the binding of nine adenine derivatives identified by docking. The same assay was employed for screening two small libraries of 69 adenosine mimics and 26 adenosine derivatives, respectively (Table 1, Figure S1).

The 69 adenosine mimics (from the Asinex screening library) were available in our laboratory from a previous project.^35^ Several members of this class of compounds feature a positively charged amine in a ring substituent of adenine. After an initial screening, 11 out of the 69 compounds were reordered from the vendor (Table S2). Three of these 11 compounds show an IC_50_ < 300 μM for METTL1 (Table 1). Furthermore, compound **5** has a very favorable ligand efficiency of 0.31 kcal/mol per non-hydrogen atom. Expectedly, since the initial library was selected to bind METTL3–14, compounds **3** and **5** show higher affinity for METTL3–14 than METTL1 (Table 1, Figures S2). On the other hand compound **4** shows slight selectivity for METTL1 against METTL3–14 and METTL16 (Table 1, Figures S2 and S3).

The 26 adenosine derivatives were selected among a series of binders of the SARS-CoV-2 mRNA Cap methyltransferases (Table S3).^36,37^ These molecules were obtained by bioisosteric replacement of the methionine moiety of SAM and several of them feature a negatively charged group that mimics the carboxyl group of methionine. Five of the adenosine derivatives show an IC_50_ < 100 μM for METTL1 and the most potent of them (compound **6**) is able to stabilize both METTL1 and METTL1-WDR4 in the thermal shift assay (Figure S5A, B). On the other hand, this series of molecules generally includes a high number of heavy atoms, which translates into a low ligand efficiency. Furthermore, they show binding to off-target human methyltransferases (e.g., glycine-*N*-methyltransferase)^36,37^ because of their structural similarity with SAM. Interestingly, compounds **6**, **8**, and **11** (Table 1) show remarkable selectivity against METTL3–14 (Figure S2) and are slightly selective also against METTL16 (Figure S3).

To avoid the inclusion of false positives, we screened the compounds of Table 1 for interference. We divided the compounds based on their IC_50_ ranges and we tested a single concentration of the compound (close to the IC_50_ value) against the downstream reaction and detection components of the assay (Figure S6). The compounds that decreased by more than 30% the maximal emission (calculated in the presence of 100 μM of sinefungin, which does not interfere with the assay) were discarded as they interfere with the downstream reaction or detection enzymes of the assay. Importantly, none of the tested adenine derivatives and adenosine mimics were interferents. All the discovered interferents belong to the class of adenosine derivatives (Table S3).

### X-ray Crystallography

The METTL1-WDR4 enzymatic assay was further validated by confirming the binding of sinefungin to the cosubstrate binding site of METTL1 using a novel soakable crystal form. The crystals are obtained through cocrystallization in the presence of SAH and feature space groups *P*2_1_2_1_2_1_ or *P*4_3_2_1_2 and high resolution. The METTL1-SAH complex structure (PDB code: 7OGJ, resolution of 1.59 Å) obtained with this protocol (detailed in the methods) is currently the highest resolution structure of the protein in complex with SAH. The METTL1-sinefungin complex structure (PDB code: 7PL1, resolution of 1.85 Å) was obtained by substituting the bound SAH with sinefungin through soaking. This structure is currently the only structure of METTL1 in complex with an inhibitor.

**Figure 1:**
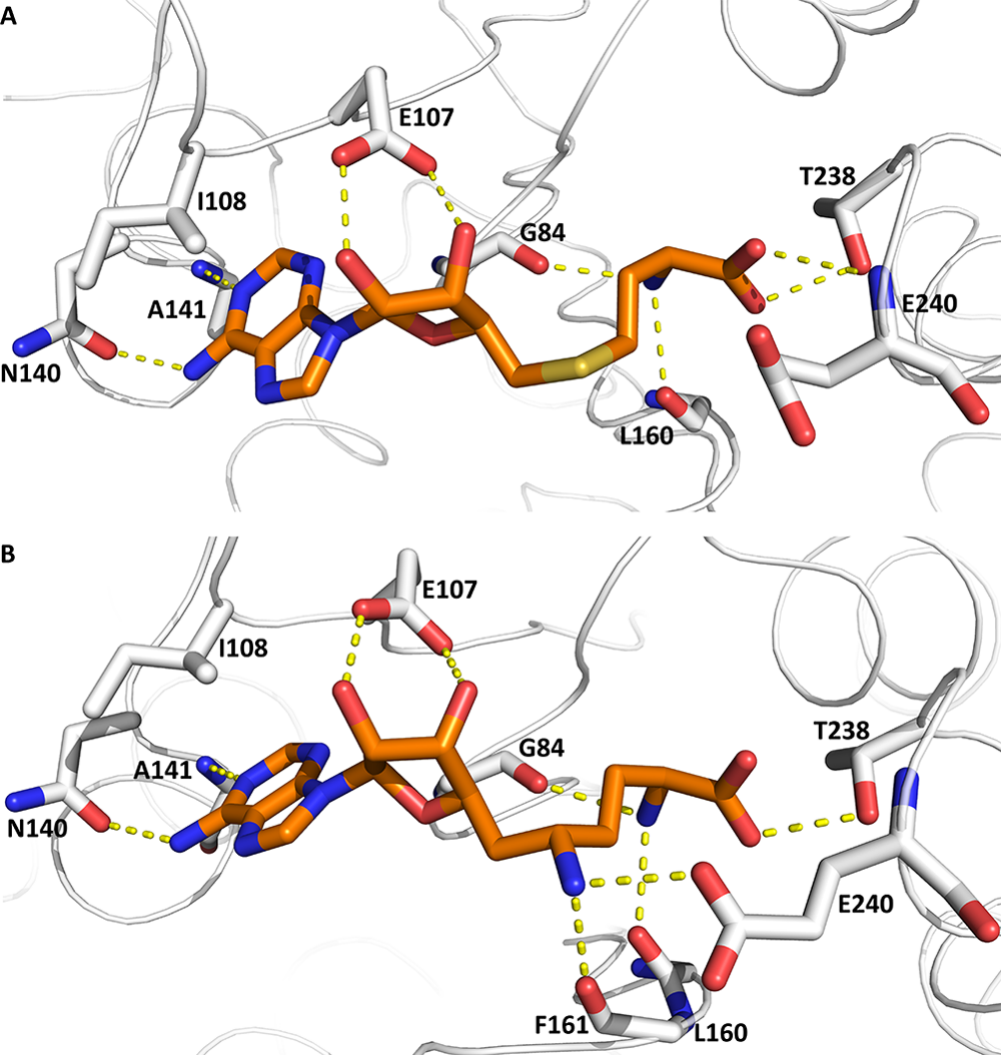
Crystal structures of the complex of METTL1 and (A) SAH and (B) sinefungin. The carbon atoms of SAH (PDB code 7OGJ) and sinefungin (PDB code 7PL1) are in orange and those of the protein in white, while hydrogen bonds are represented as yellow dashed lines.

The structure of the METTL1-SAH complex shows that the SAM binding site is rather open and exposed. Mainly polar interactions stabilize the binding of SAH. The adenine base is involved in hydrogen bonds with the side chain of N140 and the backbone NH of A141. There are also van der Waals interactions between the adenine ring and the side chain of I108. The two hydroxyl groups of the ribose interact with the side chain of E107. The methionine substructure features hydrogen bonds with the backbone carbonyls of G84, L160, the side chain of T238, and the backbone NH of E240 (Figure 1A). These interactions are highly conserved in the METTL1-sinefungin complex. In addition, the primary amine of sinefungin forms a hydrogen bond with the backbone carbonyl of F161 and a salt bridge with the side chain of E240 (Figure 1B).

While the proposed crystal form will be useful to solve the structures of METTL1 in complex with potent and soluble binders such as sinefungin, the multiple soaking attempts with the other compounds in this paper did not give positive results. The inhibitors with IC_50_ values higher than about 10 μM are not potent enough to displace the SAH bound to METTL1. As it is not possible to measure the IC_50_ value of SAH with the luminescence-based assay, we carried out a thermal shift assay. A similar shift in the melting temperature of METTL1 and METTL1-WDR4 is observed for sinefungin and SAH (Figure S7A, B). Thus, a low micromolar affinity of SAH for METTL1 can be estimated from the IC_50_ of 9 μM for sinefungin.

Since our attempts of growing a crystal without SAH in the cosubstrate pocket were not successful, the binding mode and kinetic (i.e., structural) stability of the three most ligand efficient compounds (the adenine derivatives **1**, **2**, and **5**) and the most potent inhibitor (adenosine derivative **6**) were analyzed by multiple independent MD simulations for a total sampling of 1.6 μs for each of compounds **1**, **2**, and **6**, and 3.2 μs for compound **5**.

### MD simulations

**Figure 2:**
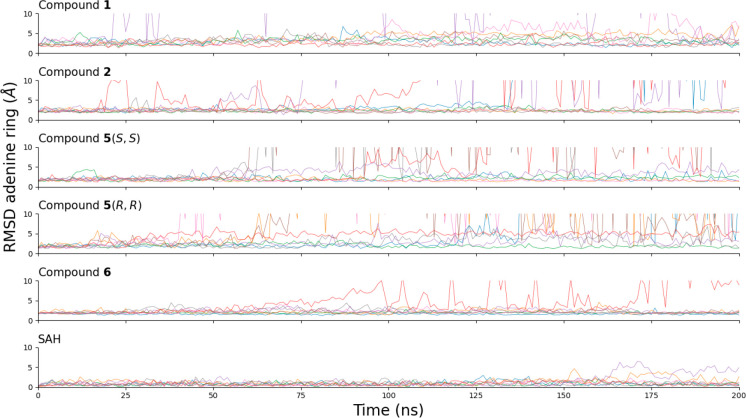
Time series of the root-mean-square deviation (RMSD) of the five compounds and SAH, which is used as a control, in the SAM binding pocket of METTL1. The RMSD was calculated between the adenine ring atoms of each compound and the adenine ring of SAH in the crystal structure (PDB code: 7OGJ). For each compound, the eight independent MD runs are shown in different colors.

**Figure 3:**
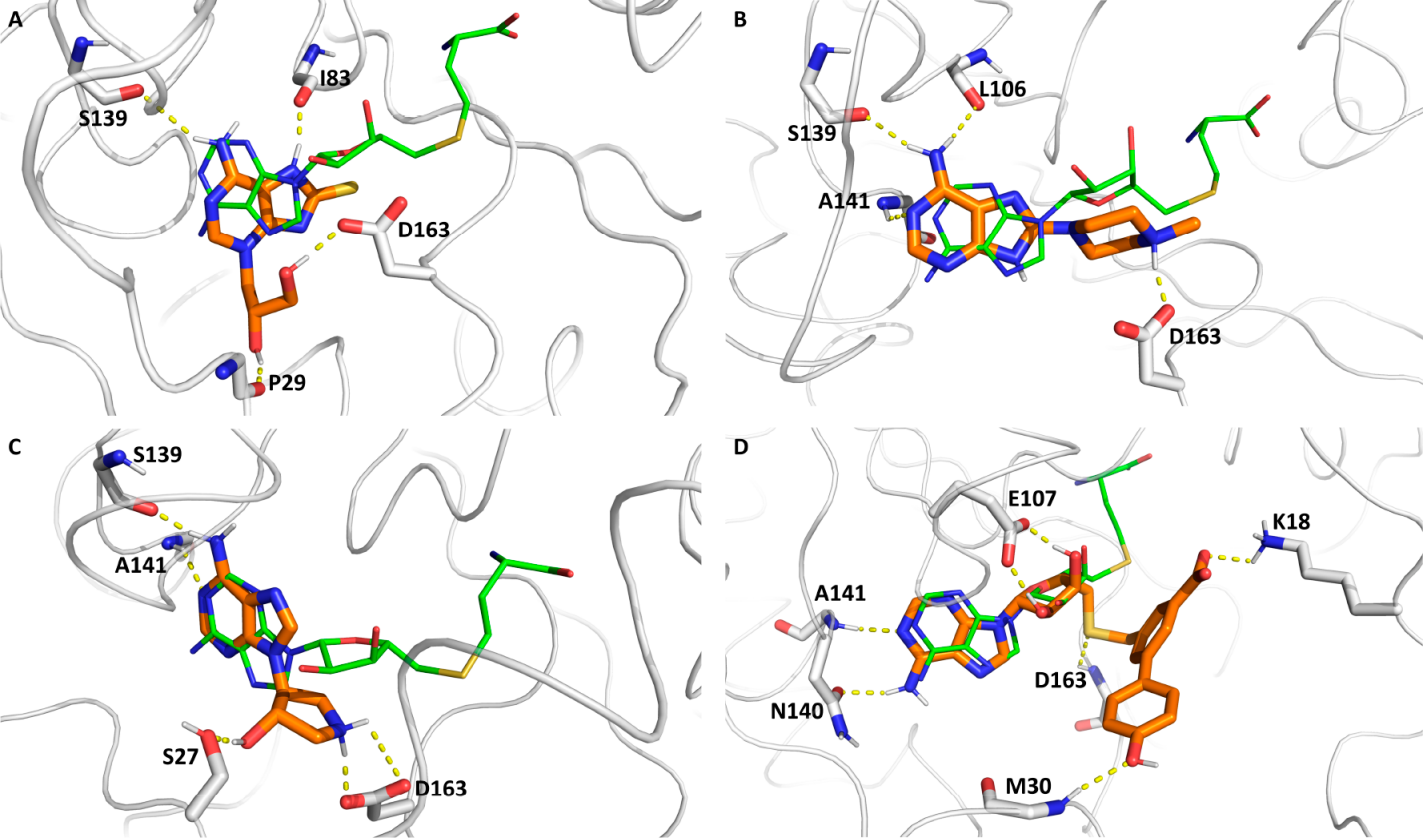
Predicted binding poses of compounds (A) **1**, (B) **2**, (C) **5**(S,S), and (D) **6**. The carbon atoms of the inhibitors are in orange and those of METTL1 in white, and hydrogen bonds and salt bridges are represented as yellow dashed lines. The binding mode of SAH (carbon atoms in green, from PDB entry 7OGJ) is shown as a basis of comparison.

For inhibitors **1**, **2**, and **6**, each enantiomer of compound **5**, and for SAH as a control, eight independent MD runs of 200 ns each were carried out. The MD simulations of compound **1** were started from the enantiomer (R) that had a favorable predicted binding energy, while the other enantiomer (S) could not be docked. Inhibitor **5** is a mixture of enantiomers where the two stereocenters C3 and C4 are in either the conformation (R,R) or (S,S). Eight independent runs were carried out for each enantiomer of compound **5**. The initial structure was obtained by minimization of the docked pose for compounds **1** and **2** while for the inhibitors **5** and **6** (which were identified *in vitro*), it was generated manually by aligning the adenine moiety to the adenine of SAH. The control MD runs for SAH were started from the crystal structure mentioned above (7OGJ). The time series of the RMSD of the adenine ring show that the predicted pose of compounds **1**, **2**, **5**(S,S), **5**(R,R), and **6** is stable (RMSD < 4 Å from the adenine in the crystal structure with SAH) in 5/8, 5/8, 5/8, 3/8 and 7/8 runs, respectively (Figure 2). The atomic coordinates of the compounds **1**, **2**, **5**(S,S), **5**(R,R), and **6** were employed for a principal component analysis and the reduced space was clustered with a Gaussian mixture model.^38^ After clustering (Figure S8) the poses representing the most populated clusters were analyzed (Figure 3). Clustering was not possible for compound **5**(R,R) because of the instability and variability of the binding pose (Figure 2 and Figure S8). This also suggests that compound **5**(S,S) may be the main one responsible for the inhibition of METTL1 and that the pure (S,S) enantiomer may feature even greater inhibitory potency than the enantiomeric mixture.

In the predicted pose of compounds **1**, **2**, and **5**(S,S) the N6 of the adenine base points toward the interior of the pocket occupied by the adenine of SAH (Figure 3A–C). The adenine ring is stabilized by hydrogen bonds with the backbone carbonyl of S139 and I83 in compound **1** (Figure 3A), and by hydrogen bonds with the backbone carbonyl of S139, and with the backbone NH of A141 in compounds **2** and **5**(S,S) (Figure 3B, C). The former additionally interacts with the backbone carbonyl of L106 (Figure 3B). The sulfur atom of **1** occupies a hydrophobic cavity lined by P162 and I83 (not shown) while the dihydroxyethyl is stabilized by hydrogen bonds with the backbone carbonyl of P29 in the N terminus, and with the side chain of D163 (Figure 3A). The piperazine ring of **2** is positioned close to the ribose of SAH and the tertiary amine is involved in a salt bridge with the side chain of D163 (Figure 3B). Similarly to compound **2**, the hydroxypyrrolidinyl group of **5** is stabilized by a salt bridge with the side chain of D163 and a hydrogen bond with the side chain of S27 (Figure 3C). In the less populated binding mode, the adenine base of compound **1** assumes an orientation that is almost perpendicular to the one of the adenine of SAH and laterally shifted toward the ribose (Figure S9A). Meanwhile, the adenine base of compound **5**(S,S) is only slightly tilted with respect to the position in the most populated binding mode and the hydroxypyrrolidinyl group assumes a perpendicular orientation to the plane of the adenine ring (Figure 3C, Figure S9B).

As expected, the adenosyl moiety of compound **6** is positioned identically to the adenosyl moiety of SAH and is stabilized by the same interactions (Figure 3C). On the other hand, the substituted biphenyl is oriented toward the solvent. The phenolic OH is involved in a hydrogen bond with the backbone NH of M30, while the benzoate forms a salt bridge with the amine of the K18 side chain (Figure 3D) and with the side chain of R109 (not shown). The pose from the second most populated cluster features an adenosyl moiety position which is laterally shifted with respect to the one of SAH while the substituted biphenyl is oriented toward the interior of the pocket (Figure S9C).

Overall, the MD simulations provide evidence that the four compounds interact with the residues lining the METTL1 cosubstrate binding pocket and can, therefore, compete with the natural binders SAM and SAH.

## Conclusions

We identified several micromolar inhibitors of METTL1 by high-throughput docking and an *in vitro* assay. Docking and force field-based energy evaluation were employed to screen a library of nearly 5000 adenine derivatives. A luminescence-based enzymatic assay was used for measuring the inhibitory activity of the top docking hits, and for screening nearly 100 adenosine derivatives/mimics. The two inhibitors identified by docking (compounds **1** and **2**) and one of the compounds identified by the *in vitro* screening (compound **5**) have very favorable ligand efficiency (0.31–0.34 kcal/mol per non-hydrogen atom). Furthermore, compound **2** is selective against the m6A-RNA methylases METTL3–14 and METTL16.

We also obtained a crystal form of SAH-METTL1 that can be used for competitive soaking and was employed to determine the structure of the complex of METTL1 with sinefungin at a resolution of 1.85 Å. Since the potency of our inhibitors is lower than that of SAH and sinefungin, we could not solve the crystal structure of their complex with METTL1. Multiple MD simulations of inhibitor **6** (IC_50_ of 41 μM) and the ligand-efficient compounds **1**, **2**, and **5** provide atomistic detail of the interactions in the cosubstrate binding pocket of METTL1. The adenine derivatives **1**, **2**, and **5** show distinct substitution patterns and have the potential as starting points for hit optimization.

## Material and Methods

### METTL1 Purification

The plasmid encoding the hexahistidine-tagged METTL1^Δ 1–31, 266–276^ construct was a gift from Cheryl Arrowsmith (Addgene ID: 25264). The protein was used to obtain the soakable crystal form and was overexpressed overnight at 18 °C in Rosetta (DE3) cells induced with IPTG at a final concentration of 200 μM. The cells were lysed using a Maximator High Pressure Homogenizer at 30 kPsi at 4 °C in a buffer composed of 50 mM HEPES (pH 7.5) and 500 mM NaCl. The lysate was clarified by centrifugation at 48 000 g at 4 °C for 1 h. The following purification steps were all performed at 4 °C. The clarified lysate was loaded on a HisTrap high performance 5 mL column (Cytiva). The loaded column was then washed with a buffer containing 50 mM HEPES (pH 7.5, 500 mM NaCl, and 40 mM imidazole. The protein was eluted using a buffer containing 50 mM HEPES pH 7.5, 500 mM NaCl, and 200 mM imidazole and dialyzed to 50 mM HEPES pH 7.5, 250 mM NaCl in the presence of TEV overnight. The protein was then diluted 20 times in a buffer containing 20 mM PIPES pH 6.5, loaded on a 5 mL HiTrap SP FF column (Cytiva) and eluted with a linear gradient of NaCl. The protein is normally eluted at a NaCl concentration of around 500 mM.

The same construct was used for the thermal shift assay but in that case, the protein was expressed and purified as detailed in ref (24).

To produce GST tagged METTL1, the hexahistidine-tagged METTL1^Δ1–31, 266–276^ was cloned into pGEX-6P-1 using *Bam*HI and XhoI as restriction sites. The protein was overexpressed overnight at 18 °C in Rosetta (DE3) cells induced with IPTG at a final concentration of 200 μM. The cells were lysed by sonication at 4 °C in a buffer composed of 100 mM Tris-HCl at pH 8.0 and 500 mM NaCl. The lysate was clarified by centrifugation at 48 000 g at 4 °C for 1 h. The following purification steps were all performed at 4 °C. The protein was purified by gravity flow using glutathione sepharose affinity resin. The resin was equilibrated by washing with 100 mM Tris-HCl (pH 8.0) and 500 mM NaCl. The soluble protein extract was loaded on the resin, and GST-METTL1 was eluted using 100 mM Tris-HCl pH 8.0, 500 mM NaCl, and 10 mM reduced Glutathione. The protein eluate was dialyzed overnight against 50 mM Tris-HCl, pH 8.0, and 150 mM NaCl. Later, an ion exchange was performed by loading the protein eluate in a SourceQ anion exchange chromatography column. The GST-METTL1 was eluted with a 50 mL salt gradient using 25 mM Tris-HCl pH 8.0 and 2 mM DTT as initial buffer; and 25 mM Tris-HCl, pH 8.0, 2 mM DTT, and 1 M NaCl as final buffer. Finally, the protein eluate was loaded into a size-exclusion Superdex 200 10/300 GL (Cytiva) column. The protein was purified in 50 mM HEPES (pH 7.5) and 150 mM NaCl.

### METTL1 HTRF Assay

To study the interaction between the pri-let-7e miRNA and METTL1, we established an assay based on homogeneous time-resolved fluorescence (HTRF) technology. In which the Eu3+-labeled antibody (HTRF donor) binds to the protein by recognizing the glutathione S-transferase (GST) tag. Meanwhile, the short and biotinylated pri-let-7e miRNA (sequence: biotin-5′-GGGCUGAGGUAGGAGG-3′ purchased from Microsynth AG) interacts with the HTRF acceptor XL665 that is bound to a tetrameric streptavidin protein. The binding between the biotinylated RNA and the GST tagged METTL1 brings the fluorophores in proximity to allow Förster resonance energy transfer (FRET).

To enhance the G-quadruplex formation, the RNA is treated by boiling at 95 °C for 3 min and by subsequent incubation on ice for 1 h. Later, the reaction is composed by mixing 6 nM GST-METTL1, 160 nM of biotinylated pri-let-7e miRNA, 40 nM Streptavidin-XL665 (4:1 ratio of oligonucleotide and Streptavidin-XL665) and 0.8 nM anti-GST Eu^3+^ in 50 mM HEPES, 150 mM NaCl, 5 mM DTT, 100 mM KCl, and 0.1% BSA. The mix was assembled in a 96 well-plate and incubated at room temperature for 3 h. After the incubation, the mix is transferred to a white low-volume, round-bottomed 384-well plate (Corning) before measurement. The signal was measured using a Spark plate reader (Tecan), with a 320 nm excitation filter and 620 nm (measurement 1) or 665 (measurement 2) emission filters, a dichroic 510 mirror, 75 flashes, and optimal gain determination, and applying a lag time of 100 μs and an integration time of 400 μs.

While in this case, the assay was used to demonstrate the formation of a complex between METTL1 and the shortened pri-let-7e miRNA, the presented protocol can also be applied to the competitive testing of binders of the METTL1 substrate binding site. This is done by maintaining the aforementioned concentrations of reagents and by adding the putative substrate site binder in single or multiple concentrations. The latter procedure permits obtaining an IC50 value for the putative binder.

### METTL1 Crystallization and Soaking

The METTL1 crystal form is obtained by cocrystallization with SAH. The protein at a concentration of 13 mg/mL is mixed with a large excess of crystalline SAH to create a saturated solution. The mix is then sonicated for 30 s, the nondissolved SAH is resuspended, and the mix is incubated for 5 min on ice. The process is repeated two times. The mix is then centrifuged for 10 min at 14 000 rpm at 4 °C to separate the SAH and METTL1 solution from the nondissolved SAH pellet. The supernatant is mixed in a 1:1 ratio with a solution of 0.1 M phosphate-citrate buffer pH 4.2, 0.2 M LiSO_4_, and 20% PEG 1K (Hampton Research). It is important to note that the buffer is obtained by titrating 0.4 M Sodium phosphate with 0.2 M citric acid to pH 4.2, the final concentration is calculated by summing the individual concentrations of the two components in the mix. Moreover, the crystals could not be obtained using fresh PEG 1k; indeed, the PEG 1k used in the experiment was aged for more than six months at RT. A potential explanation for this phenomenon is that aging can change the chemical properties and the pH of PEG.^39,40^ The pH of a fresh batch of PEG1K was measured to be 5, while it was 3 for the aged batch.

The structure of METTL1 in complex with sinefungin was obtained by soaking. A saturated solution of sinefungin in mother liquor was introduced in the crystal-containing drop and the solution incubated overnight.

### X-ray Diffraction Experiment and Structure Solution

The crystals were collected and flash-frozen in liquid nitrogen following a brief incubation in a cryo-protectant solution composed of 80% mother liquor and 20% Glycerol.

The X-ray diffraction experiment was performed on the X06DA beamline of the Paul Scherrer Institute’s Swiss Light Source. The diffraction data were processed using XDS, and XSCALE.^41^ The structures of METTL1 in complex with SAH (PDB code: 7OGJ) and sinefungin (PDB code: 7PL1) were solved through molecular replacement using PHASER,^42^ and the structure of METTL1 in complex with SAM (PDB code: 3CKK) or the structure of METTL1 in complex with SAH (PDB code: 7OGJ, chain A) as a search model, respectively. The search models were prepared by eliminating water molecules and SAM or SAH from the structure. Model rebuilding was performed using COOT.^43^ Model refinement was performed using Phenix.refine.^44^

### METTL1-WDR4 Complex Purification

The full-length, sequence-optimized, hexahistidine-tagged version of WDR4 (TWIST Bioscience), and the full-length version of METTL1 were cloned in pETDuet to obtain coexpression, using the restriction sites BamHI and SalI for WDR4, and NdelI and XhoI for METTL1. The protein complex was used for the enzymatic assay and was overexpressed overnight at 16 °C in Rosetta (DE3) cells induced with IPTG at a final concentration of 500 μM. The cells were lysed using a Maximator High Pressure Homogenizer at 30 kPsi at 4 °C in a buffer composed of 50 mM Tris-HCl pH 8.0, 200 mM NaCl, 10% glycerol, 5 mM β-mercaptoethanol, 1 mM PMSF and 5% Tween20. The lysate was clarified by centrifugation at 48 000 g at 4 °C for 1 h. The following purification steps were all performed at 4 °C. The clarified lysate was loaded on a HisTrap high performance 5 mL column (Cytiva). The loaded column was then washed with a buffer containing 50 mM Tris-HCl (pH 8.0,), 200 mM NaCl, 10% glycerol, 5 mM β-mercaptoethanol, 1 mM PMSF, 5% Tween20, and 40 mM Imidazole. The protein was eluted using a buffer containing 50 mM Tris-HCl at pH 8.0, 200 mM NaCl, 10% glycerol, 5 mM mercaptoethanol (β-ME), 1 mM PMSF, 5% Tween20, and 200 mM Imidazole. The eluate buffer was exchanged by concentrating and rediluting in 25 mM Tris-HCl pH 8.0, 2 mM DTT, and 60 mM NaCl. The protein complex was further purified by anion exchange chromatography using a HiTrap Q FF 5 mL column (Cytiva). The protein complex was eluted through a 50 mL salt gradient performed using 25 mM Tris-HCl at pH 8.0, 2 mM DTT, and 60 mM NaCl as initial buffer and 25 mM Tris-HCl, at pH 8.0, 2 mM DTT, and 1 M NaCl as final buffer. The eluate buffer was exchanged by concentrating and rediluting in 25 mM Tris-HCl pH 8.0, 2 mM DTT, and 60 mM NaCl. Protein eluate was further purified by using a HiTrap Heparin FF 5 mL column (Cytiva). The protein complex was eluted through a 100 mL salt gradient using 25 mM Tris-HCl pH 8.0, 2 mM DTT, and 60 mM NaCl as the initial buffer and 25 mM Tris-HCl, pH 8.0, 2 mM DTT, and 1 M NaCl as final buffer. In the chromatogram, two peaks should be expected: the first peak corresponds to the protein complex with a cleaved form of WDR4, while the second peak corresponds to the protein complex formed with a full-length form of WDR4. The second peak was collected and loaded into a size-exclusion Superdex 200 10/300 GL (Cytiva) column. For this last step, a buffer containing 25 mM Tris-HCl (pH, 8.0), 150 mM NaCl, and 2 mM DTT was used.

### METTL1-WDR4 Enzymatic Methyltransferase Assay

The described assay is based on the use of the MTase-Glo Methyltransferase Assay (Promega). To facilitate the G-quadruplex formation needed for the protein binding and enzymatic reaction, the oligonucleotide (sequence: 5′-GGGCUGAGGUAGGAGG-3′, purchased from Integrated DNA Technologies (IDT)) is initially diluted in a buffer composed of 100 mM KCl and 50 mM Tris-HCl pH 8.0 and treated by boiling at 95 °C for 3 min and by subsequent incubation on ice for 1 h. The reaction condition is then composed by mixing the METTL1-WDR4 complex at a concentration of 8 μM, SAM at 10 μM, and oligonucleotide at 5 μM in a buffer composed of 50 mM Tris-HCl pH 8.0, 50 mM NaCl, and 1 mM DTT. When testing inhibition, the small molecule of interest is also added to the mix and tested as a set of 2 or 1.5-fold dilutions where the starting concentration is normally 1 mM. During the initial screening, the small molecules were tested as a single point and the residual signal was calculated as percentage of the maximum possible signal. After assembly, the mix is then incubated for four h at 37 °C. After the incubation, the reaction buffer provided with the kit is added in a 1/4 volume ratio with respect to the reaction condition, and the mix is then incubated for 30 min at room temperature. Note that the reaction buffer can also be used in a 1/8 volume ratio without significant change in the results (i.e., the resultant IC_50_). This allows for a minor use of reagents as well as a lower background and a higher assay window. Following the 30 min incubation, the detection solution provided with the kit is added in 1/4 volume ratio to the mix which is again incubated for 30 min at room temperature. The mixture is transferred in a white, low-volume, 384-well plate (Corning). The luminescence signal is measured using a Tecan Spark plate reader and its standard luminescence acquisition protocol: 1000 ms of integration time, 0 ms of settle time, no attenuation, and counts/s output format. The IC_50_ values are determined using GraphPad Prism 9.5.1 Nonlinear regression fit [Inhibitor] vs response – Variable slope (four parameters).

When interference was tested, the METTL1-WDR4 complex and the oligonucleotide are excluded from the mix. In order to simulate SAH produced during the methyltransferase reaction, SAH is supplied artificially. The concentration of SAH normally produced during the methyltransferase reaction was estimated through a SAH titration curve (as indicated by the manufacturer) to be 0.5 μM. To better simulate the reaction conditions, this amount of SAH was subtracted from the concentration of SAM normally used in the reaction.

### Analytical Gel Filtration of METTL1-WDR4 and Pri-let-7e

The 120 bp version of pri-let-7e (sequence: 5′-CUGUCCACCUGCCGCGCCCCCCGGGCUGAGGUAGGAGGUUGUAUAGUUGAGGAGGACACCCAAGGAGAUCACUAUACGGCCUCCUAGCUUUCCCCAGGCUGCGCCCUGCACGGGACGGGG-3′ purchased form IDT) is initially diluted in a buffer composed by 50 mM Tris-HCl pH 8.0, 150 mM NaCl, and 50 mM KCl, boiled at 95 °C for 3 min and subsequently incubated on ice for 1 h. The treated RNA is then mixed with METTL1-WDR4 at a final concentration of 4 μM and, 6 μM, respectively (1:1.5 ratio) in the aforementioned buffer. The mix is incubated on ice for 45 min and subsequently at room temperature for 15 min. The mix is then centrifuged for 10 min at 14 000 rpm, 4 °C and loaded into a size-exclusion Superdex 200 10/300 GL (Cytiva) column. The fraction corresponding to the tripartite complex is isolated. In order to test the stability of the tripartite complex, the fraction is incubated for 30 min at 4 °C and loaded again into the size-exclusion Superdex 200 10/300 GL column.

### METTL3–14 Expression, Purification, and HTRF Assay

The expression and purification of METTL3–14, as well as the HTRF assay, were carried out as described before.^45^ The IC_50_ values are determined using GraphPad Prism 9.5.1 Nonlinear regression fit [Inhibitor] vs response – Variable slope (four parameters).

### METTL16 Purification

The full length hexahistidine-tagged version of METTL16 was cloned in pETDuet-1, using the *Bam*HI and XhoI restriction sites. The protein construct was overexpressed overnight at 18 °C in Rosetta (DE3) cells, induced with IPTG at a final concentration of 500 μM. The cells were lysed using a Maximator High Pressure Homogenizer at 30 kPsi at 4 °C in a buffer composed of 25 mM Tris-HCl, pH 7.5, 500 mM NaCl, 5% v/v glycerol, 0,5% Tween 20 and 0,5 mM TCEP. The lysate was clarified by centrifugation at 48 000 g at 4 °C for 1 h. The clarified lysate was loaded in a Ni-NTA affinity column (5 mL of HisTrap FF from GE Healthcare). The loaded column was then washed with a buffer containing 25 mM Tris-HCl, pH 7.5, 500 mM NaCl, 5% v/v glycerol, 0,5% Tween 20, 0,5 mM TCEP, and 50 mM Imidazole. The protein was eluted using a buffer containing 25 mM Tris-HCl, pH 7.5, 500 mM NaCl, 5% v/v glycerol, 0,5% Tween 20, 0,5 mM TCEP, and 250 mM Imidazole. The eluate buffer was exchanged by concentrating using a centrifugal filter and rediluting in 25 mM Tris-HCl pH 7.5 and 50 mM NaCl. The protein was further purified by anion exchange chromatography using a HiTrap Q-HP column (5 mL column volume, GE Healthcare). The protein was eluted through a 50 mL salt gradient performed using 25 mM Tris-HCl pH 7.5, 50 mM NaCl as initial buffer, and 25 mM Tris-HCl pH 7.5, 1 M NaCl as final buffer. In the chromatogram, two peaks should be expected: the first peak corresponds to a cleaved form of METTL16, and the second peak corresponds to the full-length protein. Finally, the second peak was collected, loaded into a size-exclusion Superdex 200 10/300 GL (Cytiva) column, and purified with 20 mM Tris-HCl, pH 8.0, and 150 mM NaCl.

### METTL16 Enzymatic Assay

Similar to the case for METTL1-WDR4, we established and optimized an enzymatic assay for the screening of binders of METTL16. The assay is also based on the use of the MTase-Glo Methyltransferase Assay and exploits the full-length METTL16 and the modified version of its natural substrate MAT2A hairpin 1 G20 → A20^46^ with sequence 5′-UGUUGGCGUAGGCUACAGAAAAGCCUUCAAG-3′ (Microsynth AG). The reaction is composed by mixing METTL16 at a concentration of 4 μM, SAM at 10 μM, and oligonucleotide at 10 μM in 50 mM Tris-HCl pH 8.0, 50 mM NaCl, and 1 mM DTT. The mix is incubated for two h at 37 °C. After the incubation, the reaction buffer and detection solution provided by the kit are added as described for the METTL1-WDR4 enzymatic assay. Finally, the mixture is transferred to a white, low-volume, 384-well plate (Corning). The luminescence signal is measured using a Tecan Spark plate reader in its standard luminescence acquisition protocol described previously. The IC50 values are determined using GraphPad Prism 9.5.1 Nonlinear regression fit [Inhibitor] vs response-variable slope (four parameters).

### Thermal Shift Assay

The interaction between compound **6**, SAH, sinefungin, shortened pri-let-7e (sequence: 5′-GGGCUGAGGUAGGAGG-3′), 120bp pri-let-7e (sequence: 5′-CUGUCCACCUGCCGCGCCCCCCGGGCUGAGGUAGGAGGUUGUAUAGUUGAGGAGGACACCCAAGGAGAUCACUAUACGGCCUCCUAGCUUUCCCCAGGCUGCGCCCUGCACGGGACGGGG-3′) and METTL1 and the one between compound **6**, shortened and 120 bp pri-let-7e, and METTL1-WDR4 were monitored through thermal shift assay. The proteins were buffered in 50 mM Tris-HCl at pH 8.0, 50 mM NaCl, and 1 mM DTT and assayed in a 96 well plate at a final concentration of 2 μM (with the only exclusion of METTL1-WDR4 vs compound **6**, where the protein was tested at a concentration of 0.5 μM). SYPRO Orange dye was added to the mix with a volume ratio of 1:1000. The molecule of interest is also added to the mix and tested as a set of 2-fold dilutions where the starting concentration is normally 1 mM or at single concentration. The fluorescence monitoring was performed using a LightCycler 480 System. The temperature was set up to increase with a ramp rate of 0.06 °C/s from 20 to 85 °C and 10 acquisitions per °C were taken in dynamic integration time mode and using red 610 (498–610) filter combination. The melting curves were calculated using the Tm calling analysis of LightCycler 480 software release 1.5.1.62 SP3.

### Fragment Docking and Selection

A library of 4896 small molecules was considered for in silico screening. These molecules were selected from the Zinc2020 database so that they had a number of non-hydrogen atoms between 15 and 23 and an adenine ring in the structure.

The structure of METTL1 used for docking is the one in the complex with S-adenosyl-l-homocysteine (PDB code: 7OGJ). The binding site for SEED docking consisted of only A141. The partial charges and van der Waals parameters for the atoms in the protein and the small molecules were taken from the CHARMM36 all-atom force field^47,48^ and the CHARMM general force field (CGenFF), respectively.^47−49^ Importantly, the CHARMM36 force field and CGenFF are fully consistent in their partial charges and van der Waals parameters. The evaluation of the binding energy in the program SEED^29,32^ consists of a force field-based energy function with a continuum dielectric approximation of desolvation penalties by the generalized Born model.^31^ The values of the dielectric constant were 2.0 and 78.5 for the volume occupied by the solute and solvent, respectively. The compounds were ranked according to the binding energy calculated by SEED. Nine compounds were finally purchased based on commercial availability and structural diversity.

### Molecular Dynamics Simulations

Multiple MD runs were started for METTL1 in complex with the compounds and SAH. The METTL1 structure was obtained from the PDB (code: 8EG0),^6^ using residues 12 to 265. The SAH pose was obtained by aligning the structure in complex (PDB code: 7OGJ) to 8EG0. The initial position of the compounds was obtained either from the lowest-energy docked pose (compounds **1** and **2**) or by aligning their adenine moiety to the SAH adenine (compounds **5** and **6**). All simulations were performed with GROMACS 2021.5^50^ using the CHARMM36m force field.^51^ All tested compounds were parametrized using CGenFF force field version 4.6 (ref.^49^) using the web interface (version 1.0.0). The version of the force field (4.6) is consistent with the CGenFF version included in the CHARMM36m force field. The complex was solvated in an 89 Å cubic box of water molecules with added Na^+^ and Cl^–^ ions at a concentration of 150 mM. Afterward, the system was subjected to energy minimization. A 5 ns NVT equilibration was performed, in which the system was kept under positional restraints to reach 300 K. For the manually aligned compounds (**5** and **6**) the positional restraints were applied only to the adenine ring atoms. For the production MD, eight independent copies of each complex were run for 200 ns. In the experimental sample, compound **5** is a mixture of the (R,R) and (S,S) enantiomers; therefore, eight independent copies were run for each of them. The RMSD of the compounds with respect to the adenine ring of SAH was monitored throughout the sampling for each of the eight runs.

The calculation of representative poses was done by clustering of compound coordinates. Coordinates were aligned by using C_α_ atoms of the protein as reference. Frames were considered as having a bound compound if the RMSD to the SAH adenine was less than 4 Å. For all frames after 100 ns of simulation, the coordinates of the bound compound were extracted. A principal component analysis (PCA) of the atomic coordinates for all of the bound frames was used to reduce the dimensionality of the Cartesian coordinate space. A Gaussian mixture model was used to assign cluster identity to the frames in the reduced space. A single cluster contained most of the bound frames, except for compounds **1**, **5**(S,S), and **6** where a second cluster had also a considerable number of structures. The centroid was defined as the data point (frame) with the highest modeled probability density for each cluster. The frames containing the centroid of each cluster were output and considered as representative poses. For compounds **1**, **5**(S,S), and **6** a second cluster centroid was analyzed. Analyses were done using the Python package mdtraj,^52^ while dimensionality reduction and clustering used scikit-learn.^38^

### Compound Source and Purity

The adenine derivatives were purchased from Mcule and have guaranteed purity >90%. The adenosine mimics were purchased from Asinex. The adenosine derivatives were provided by Dr. Olga Bobileva (Latvian Institute of Organic Synthesis, Riga) and their synthesis is described in ref.^36,37^ The purity of the adenosine derivatives was confirmed to be >90% with Waters Alliance LC systems equipped with 2695 separation module with LiChrospher PR Select 4.0 × 250 mm or Apollo 5 μm C18 4.6 × 150 mm column and Waters 2489 dual absorbance detector. Gradient 0–100% over 15 min; solvent A: 5% acetonitrile in 0.1% H_3_PO_4_; solvent B: 95% acetonitrile in 0.1% H_3_PO_4_; flow rate: 1 mL/min; column temperature: 40 °C. The HPLC traces of the adenosine derivatives of Table 1 are reported in the Supporting Information.

### Safety Statement

No unexpected or unusually high safety hazards were encountered.

## Supporting Information Available

Tables of the tested adenine and adenosine derivatives; dose–response curves for METTL1-WDR4, METTL3–14, METTL16, and the 11 compounds; proofs of pri-let-7e and METTL1/METTL1-WDR4 complex formation; thermal shift of METTL1 and METTL1-WDR4 in the presence of compound **6**; interference screening of the compounds; dose–response thermal shift of METTL1 and METTL1-WDR4 in the presence of SAH and sinefungin; calculation of representative binding poses for the last 100 ns of each independent simulation; secondary binding modes of **1**, **5**(S,S), and **6** as observed in the MD simulations; data collection and refinement statistics for 7OGJ and 7PL1; and HPLC traces of compounds 6–11 (PDF)
Initial structures used for molecular dynamics simulations (ZIP)

## Accession Codes

The atomic coordinates and structure factors for the crystal structures of METTL1 in the complex with SAH and sinefungin have been released in the Protein Data Bank (PDB) with the accession codes 7OGJ and 7PL1, respectively. METTL1 UniProt accession ID: Q9UBP6. WDR4 UniProt accession ID: P57081. METTL16 UniProt accession ID:Q86W50. METTL3 UniProt accession ID: Q86U44. METTL14 UniProt accession ID: Q9HCE5.
