# Supplementary material for: Small-Molecule Inhibitors of the m7G-RNA Writer METTL1
Source: ACS Bio Med Chem Au. 2023 Dec 12;4(2):100–10. doi: 10.1021/acsbiomedchemau.3c00030 (PMC11027120; doi:10.1021/acsbiomedchemau.3c00030)
Supplement: Supplementary file 2 — bg3c00030_si_002.pdf [file bg3c00030_si_002.pdf]

Small-molecule inhibitors of the m[7]G-RNA writer METTL1

Francesco Nai[a], Maria Paula Flores Espinoza[a], Annalisa Invernizzi[a], Pablo Andrés Vargas Rosales[a], Olga Bobileva[b], Marcin Herok[a], and Amedeo Caflisch[a,*]

[a]Department of Biochemistry, University of Zurich, Winterthurerstrasse 190, CH-8057 Zurich, Switzerland
[b]Latvian Institute of Organic Synthesis, Aizkraukles 21, Riga LV-1006, Latvia
[*]To whom correspondence should be addressed. Tel: +41 44 635 5521; Email: caflisch@bioc.uzh.ch

Supporting information

Compound number	2D structure	SEED energy (rank)	Residual signal at 1 mM compound concentration (%)	IC50 M1-WDR4 (uM)	HillSlope
	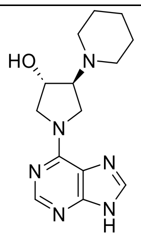	-17.43 (7)	98		
	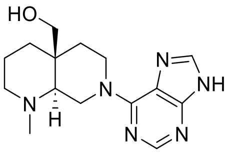	-25.32 (1)	76		
	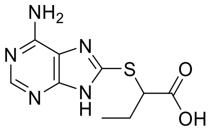	-17.19 (8) [S]	80		
	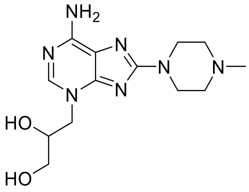	-16.04 (25) [S]	89		
**1**	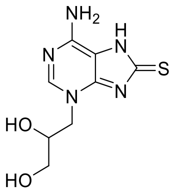	-18.10 (5) [R]	50	144	-0.97

	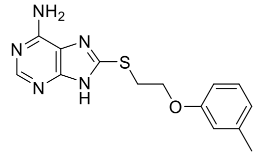	-16.65 (17)	102		
	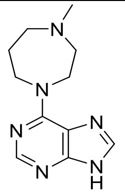	-18.67 (3)	82		
**2**	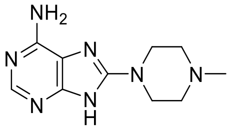	-17.56 (6)	46	187	-0.91
	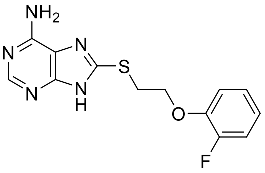	-17.16 (10)	94		

Table S1. *In vitro* characterization of the nine adenine derivatives identified by the docking campaign on METTL1. The SEED energy is the predicted binding energy in kcal/mol. Where present, the IC50 value is the average of two or more biological replicates, and each biological replicate is the average of two technical replicates. In case of enantiomeric compounds, the enantiomer to which the SEED energy and rank are referred to is specified in square parenthesis.

Compound number	ASINEX code	Structure	Residual signal at 2.5 mM compound concentration (%)	IC50 M1-WDR4 (μM)	HillSlope
	LAS51494822	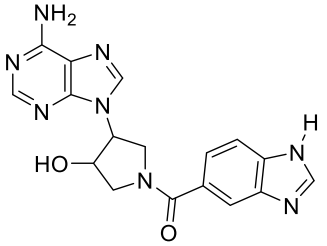	58		
	LAS 40775464	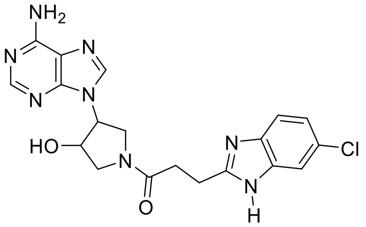	40	>1000	-2.00

	BDG34071792	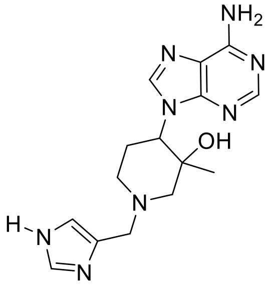	20	478<br>549	-1.35<br>-1.34
	BDH34001749	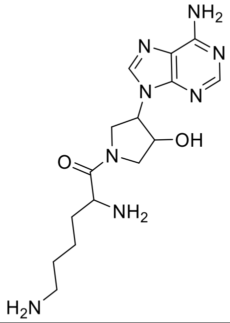	28	960<br>586	-1.26<br>-1.40
	BDH33909967	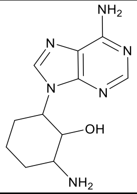	10	473<br>555	-1.09<br>-1.11
**5**	LAS33909600	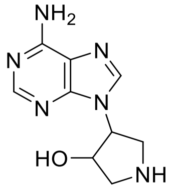	1	277	-1.45
	BDG33904788	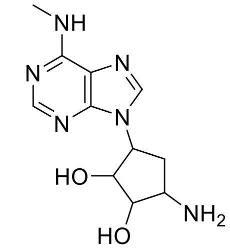	21	783<br>634	-1.91<br>-1.91
**4**	BDH33920757	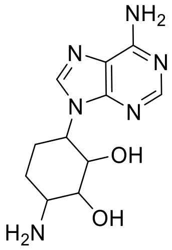	8	212	-1.11
	BDG34073059	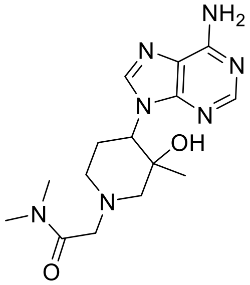	68		

	LAS34159697	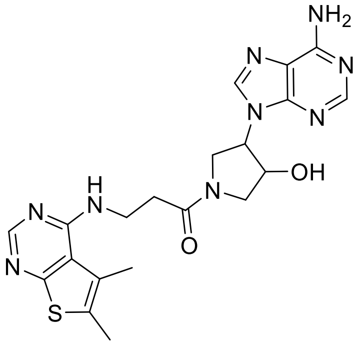	7	291<br>271	-1.40<br>-1.26
**3**	BDG34159698	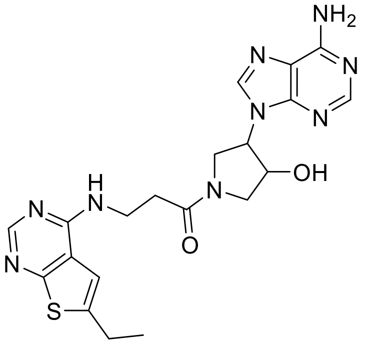	-1	164	-1.24

Table S2. *In vitro* characterization of the 11 compounds selected after the initial METTL1-WDR4 enzymatic assay screening of the library of adenosine mimics. When present, the IC50 values are from the average of two technical duplicates, except for the compounds reported in the main text (numbered) for which we report the average of at least two biological replicates, and each biological replicate is the average of two technical replicates.

Compound number	Structure	Residual signal at 1 mM compound concentration (%)	IC50 M1-WDR4 (µM)	HillSlope
	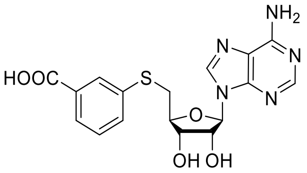	47		
	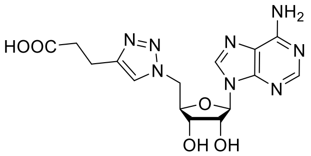	323		
	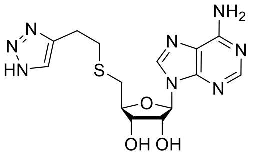	82		

**11**	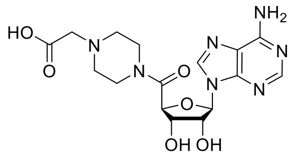	13	178	-1.01
	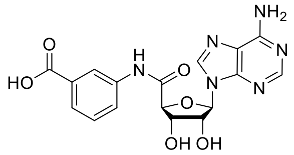	38	>1000	-1.60
	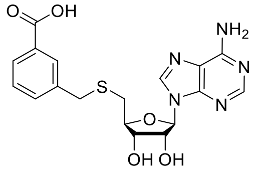	55		
	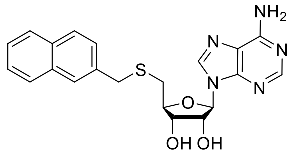	14		
	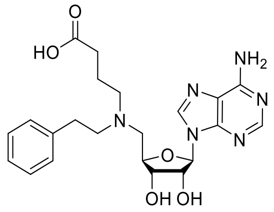	28	>1000	-1.06
	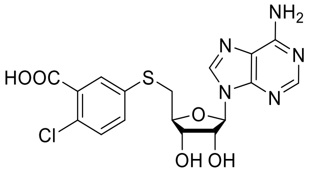	30	608<br>579	-1.56<br>-0.98
	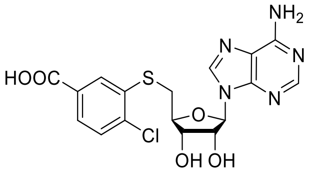	26	419<br>311	-1.04<br>-1.21
	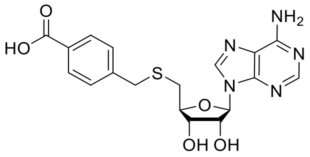	28	393<br>562	-0.98<br>-1.33
**8**	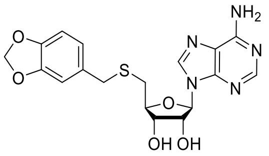	16	52	-1.22
	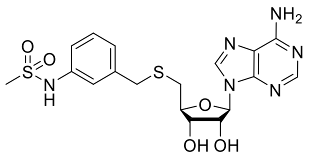	4	Interferent:<br>reduces the<br>maximal signal to<br>44% at 400 µM	

	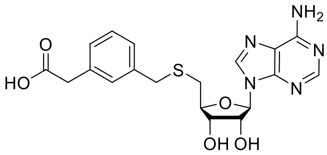	28	423<br>413	-1.21<br>-1.92
	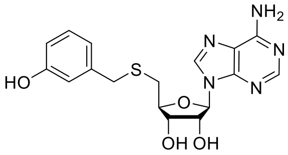	51		
	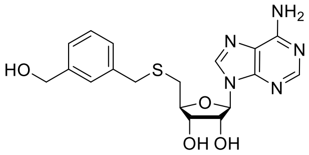	6	Interferent:<br>reduces the<br>maximal signal to<br>69% at 100 μM	
	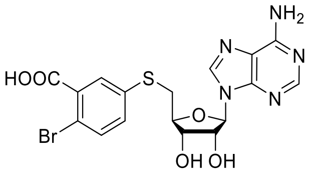	30		
	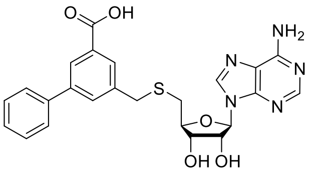	18	Interferent:<br>reduces the<br>maximal signal to<br>50% at 20 μM	
**9**	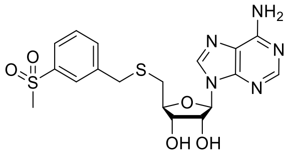	16	61	-1.03
	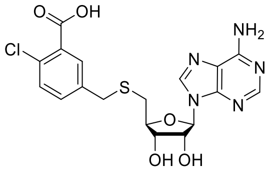	24		
	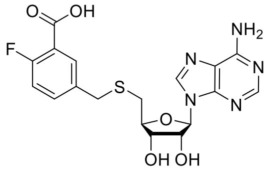	32	312<br>507	-1.19<br>-1.02
	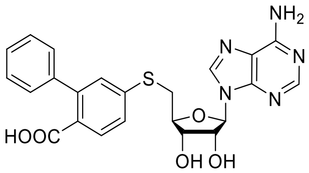	9	280<br>319	-1.16<br>-0.81

	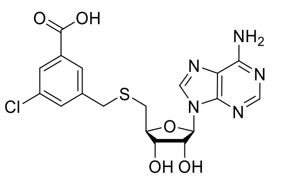	8	Interferent: reduces the maximal signal to 65% at 200 µM	
**10**	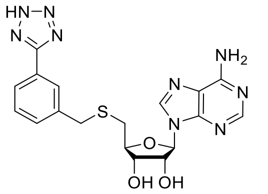	11	78	-1.09
**6**	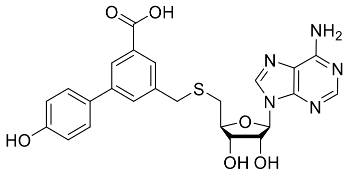	6	41	-1.01
**7**	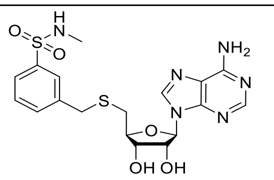	12	47	-1.23

Table S3. Same as Table S2 for the 26 adenosine derivatives screened using the METTL1-WDR4 enzymatic assay. For the compounds with residual signal at 1 mM compound concentration (%) in red the IC50 value could not be calculated.

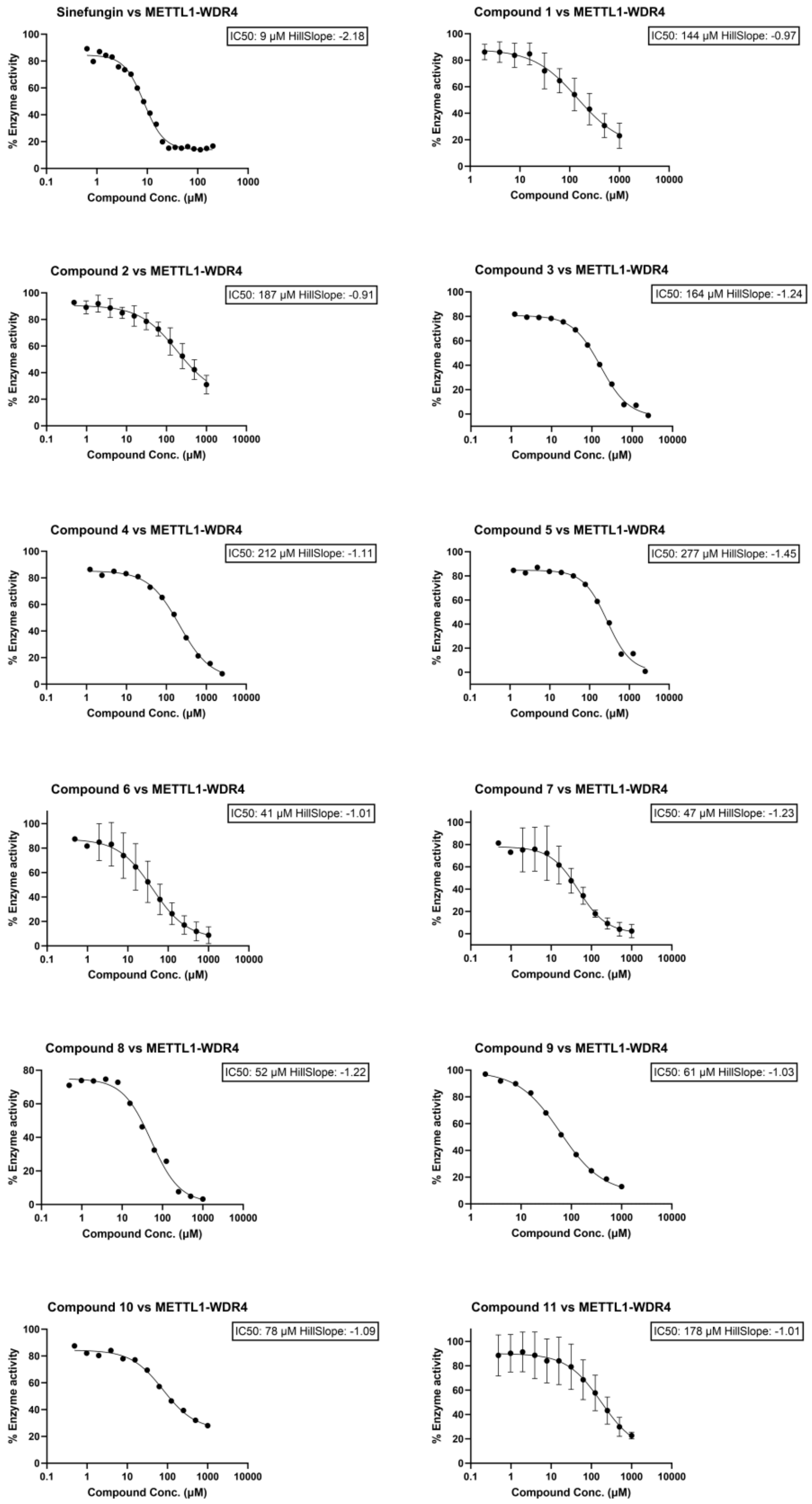

Figure S1. Dose-response curves for METTL1-WDR4 and the 11 compounds presented in Table 1. The curves come from the average of two or more biological replicates, and each biological replicate is the average of two technical replicates. The error bars represent the standard deviation for the biological triplicates.

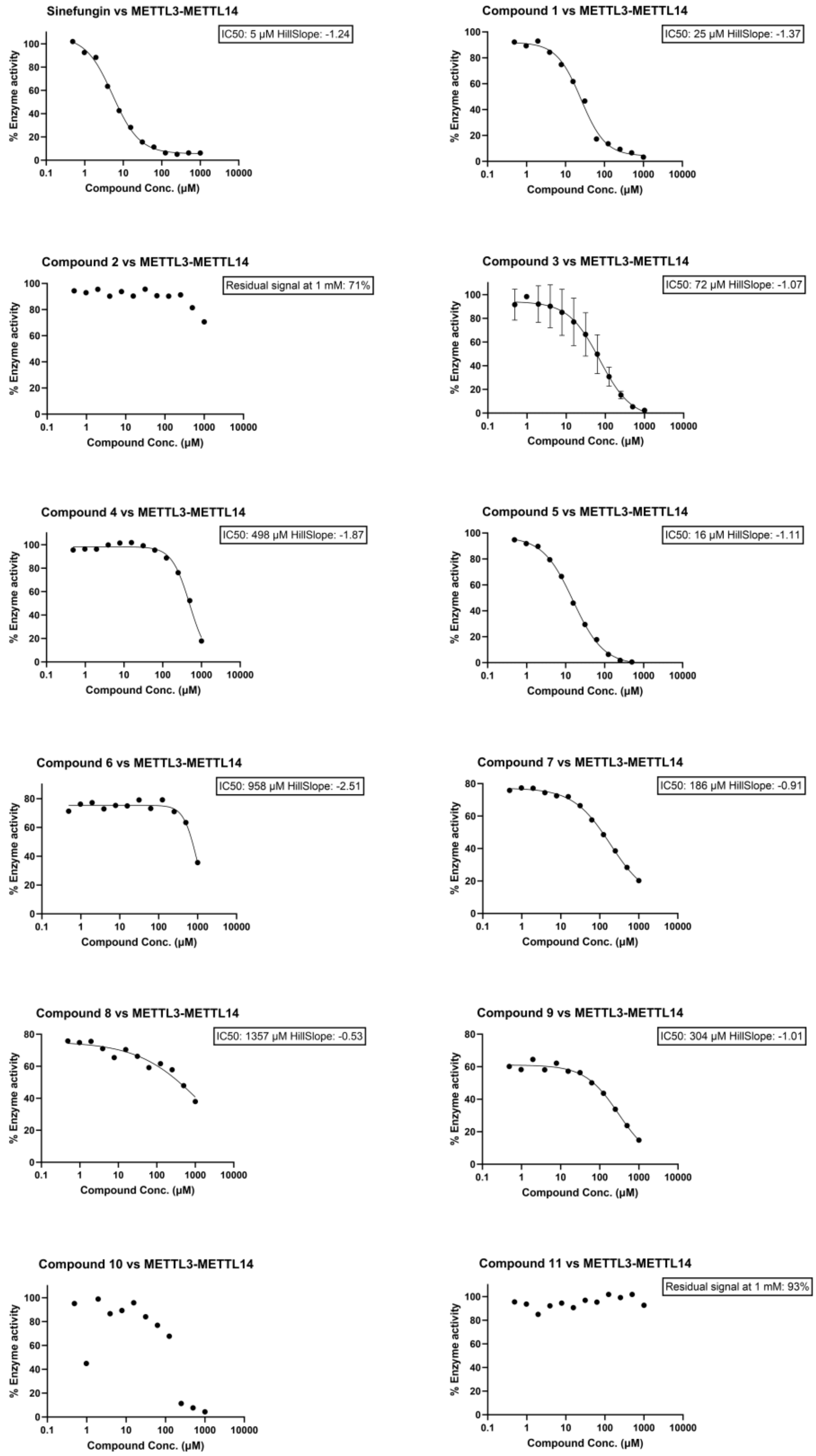

Figure S2. Selectivity testings against METTL3-METTL14 for the 11 compounds presented in Table 1. The curves come from the average of two or more biological replicates, and each biological replicate is the average of two technical replicates. The error bars represent the standard deviation for the biological triplicates.

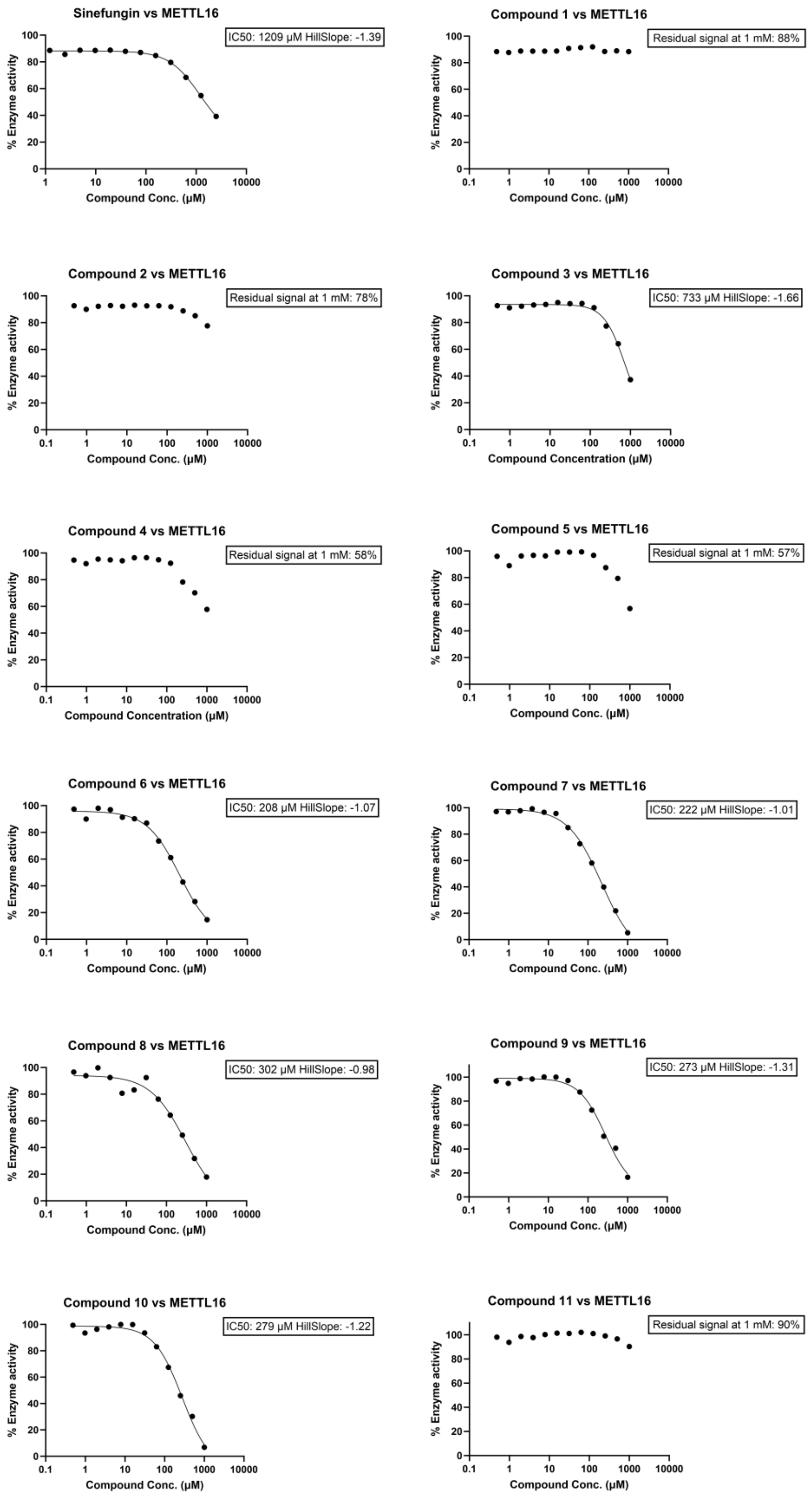

Figure S3. Selectivity testings against METTL16 for the 11 compounds presented in Table 1. The curves come from the average of two biological replicates, and each biological replicate is the average of two technical replicates.

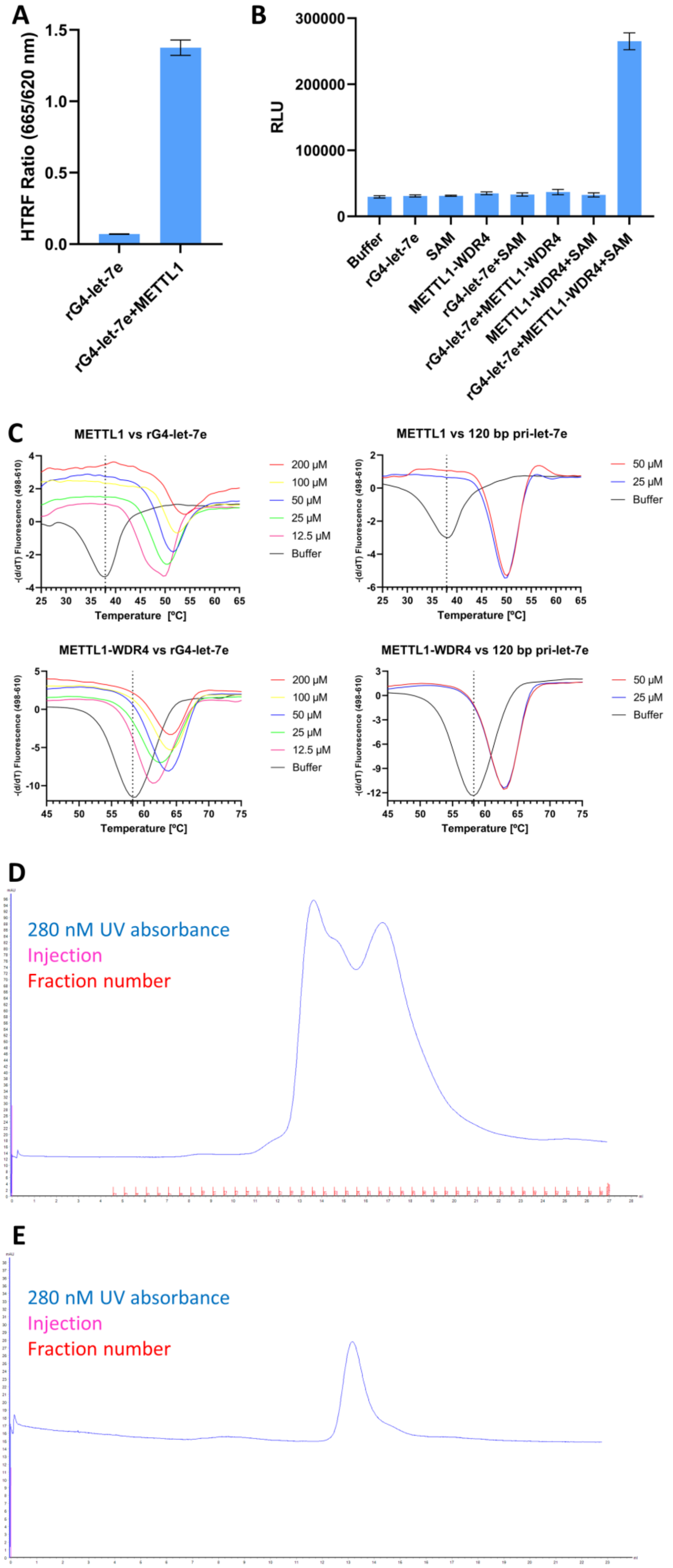
A
HTRF Ratio (665/620 nm)
1.5
1.0
0.5
0.0
rG4-let-7e
rG4-let-7e+METTL1
B
RLU
300000
200000
100000
0
Buffer
rG4-let-7e
SAM
METTL1-WDR4
rG4-let-7e+SAM
rG4-let-7e+METTL1-WDR4
METTL1-WDR4+SAM
rG4-let-7e+METTL1-WDR4+SAM
C
METTL1 vs rG4-let-7e
-(d/dT) Fluorescence (498-610)
Temperature [ºC]
200 μM
100 μM
50 μM
25 μM
12.5 μM
Buffer
METTL1 vs 120 bp pri-let-7e
50 μM
25 μM
Buffer
METTL1-WDR4 vs rG4-let-7e
METTL1-WDR4 vs 120 bp pri-let-7e
D
280 nM UV absorbance
Injection
Fraction number
E
280 nM UV absorbance
Injection
Fraction number

Figure S4. pri-let-7e and METTL1/METTL1-WDR4 complex formation. (A) FRET emission in presence of rG4-let-7e or rG4-let-7e+METTL1. (B) Luminescence emission from the METTL1 enzymatic assay in presence of different combinations of the components. A significant luminescence emission can only be observed when both METTL1-WDR4 and the rG4-let-7e are added to the reaction. (C) Thermal shift of METTL1 and METTL1-WDR4 in presence of rG4-let-7e or 120bp pri-let-7e. (D) Size exclusion profile of METTL1-WDR4 in presence of 120 bp pri-let-7e on a Superdex 200 10/300 GL column (Cytiva). The elution volumes of the first peak correspond to the molecular weight of the pri-let-7e-METTL1-WDR4 complex while the ones of the second and third peaks correspond to the molecular weight of METTL1-WDR4 and pri-let-7e, respectively. (E) Size exclusion profile after reinjection of the first peak on a Superdex 200 10/300 GL column (Cytiva), the tripartite complex does not dissociate.

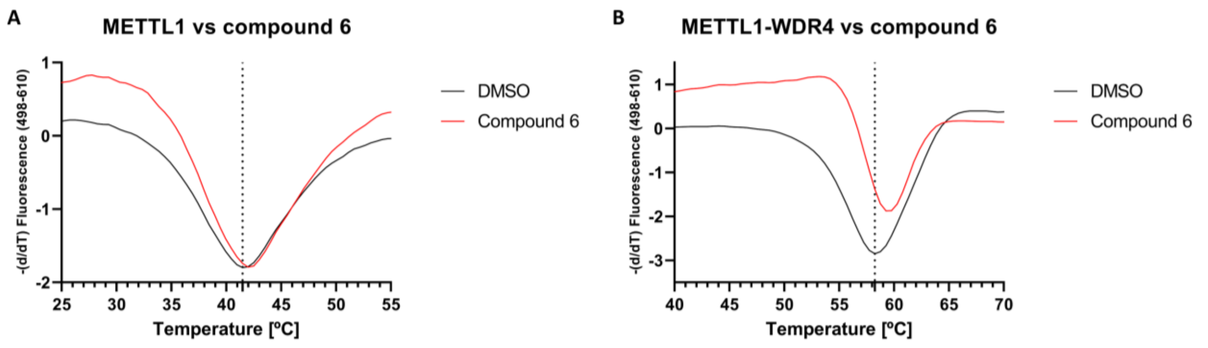

Figure S5. Thermal shift of METTL1 (A) and METTL1-WDR4 (B) in the presence of DMSO control (black) or compound **6** (red) which causes thermal stabilization at 1 mM concentration.

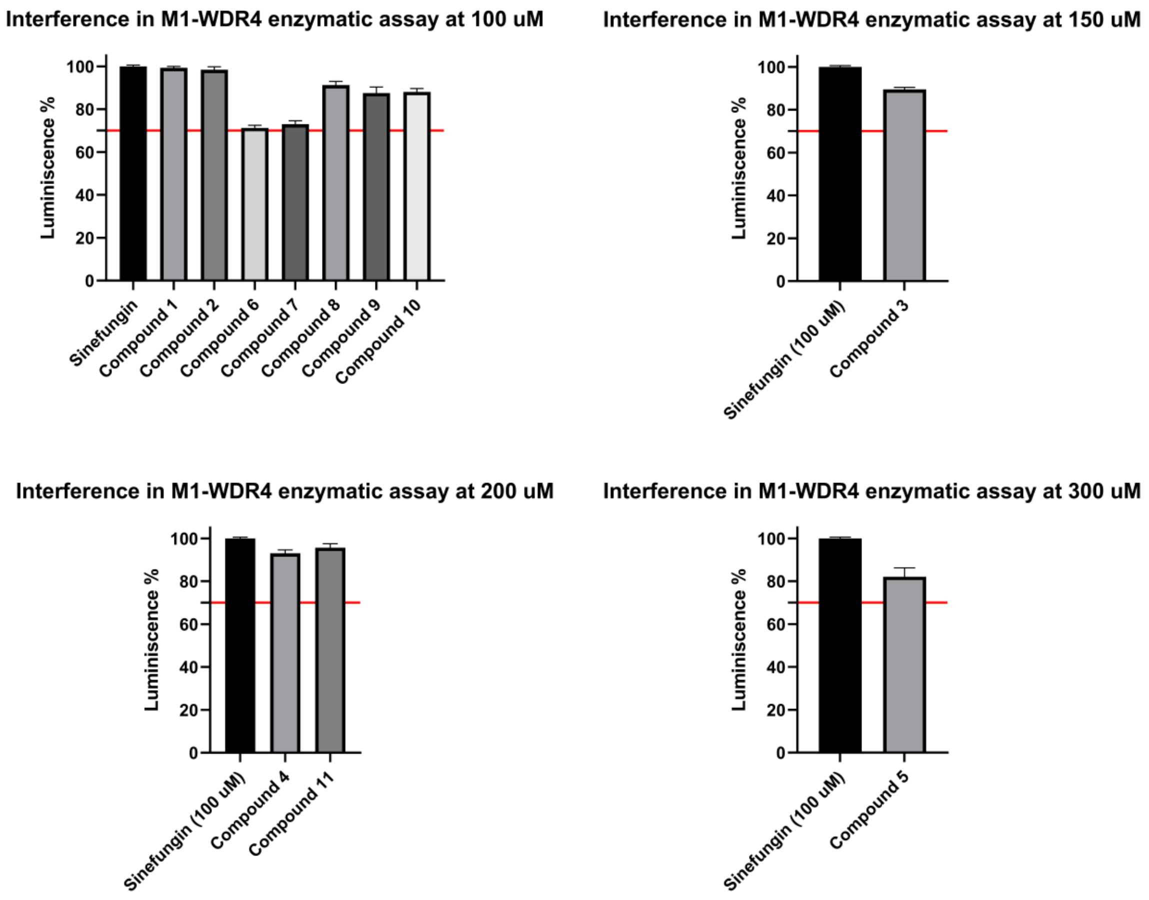

Figure S6. Interference screening of the compounds. The compounds that decreased by more than 30% the maximal emission (calculated in presence of 100 µM of sinefungin, which does not interfere with the assay) were excluded. The threshold at 70% is indicated as a red line. The interfering compounds are indicated in Table S3.

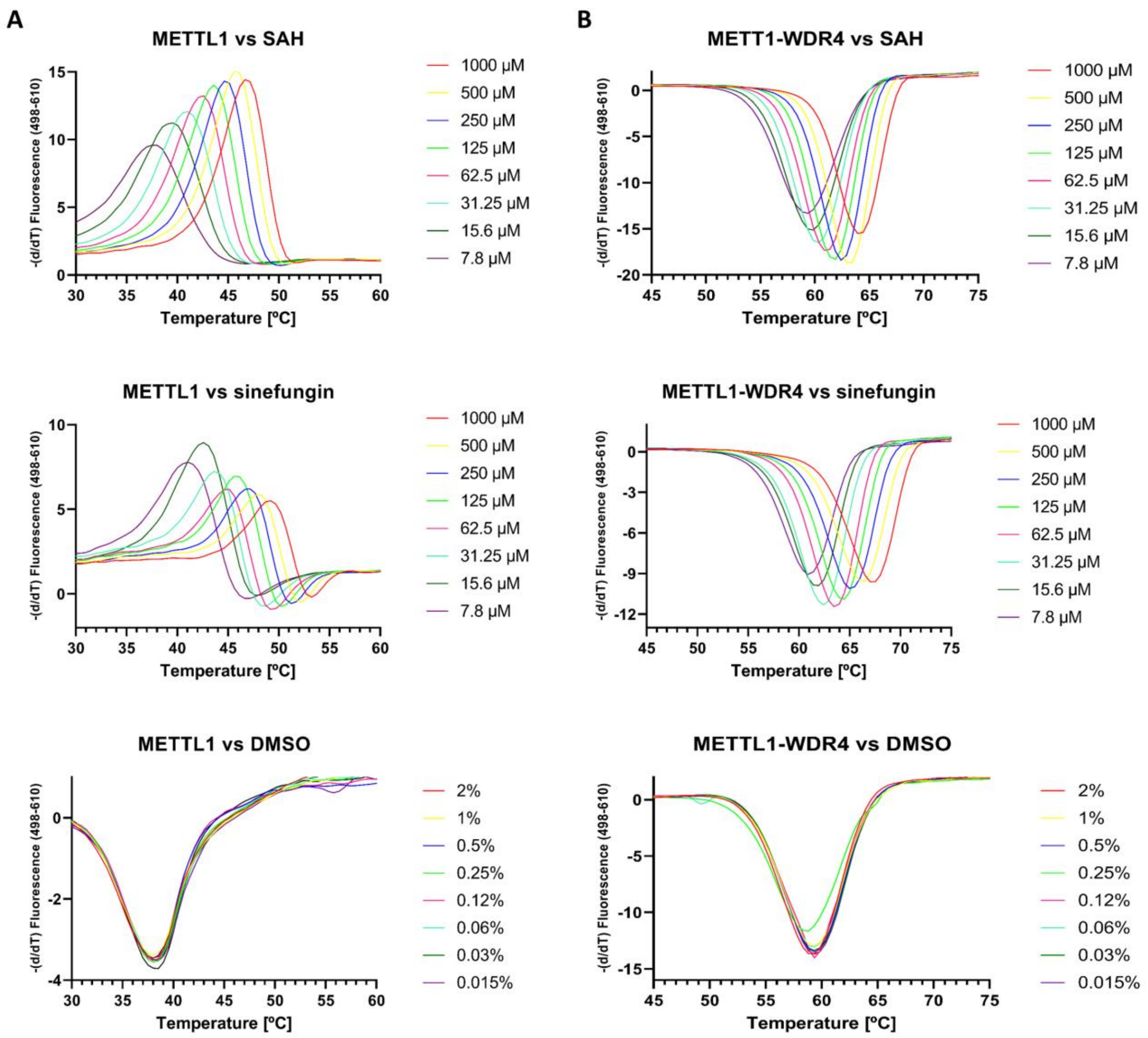

Figure S7. Dose-response thermal shift of METTL1 (A) and METTL1-WDR4 (B) in the presence of SAH, sinefungin, and DMSO.

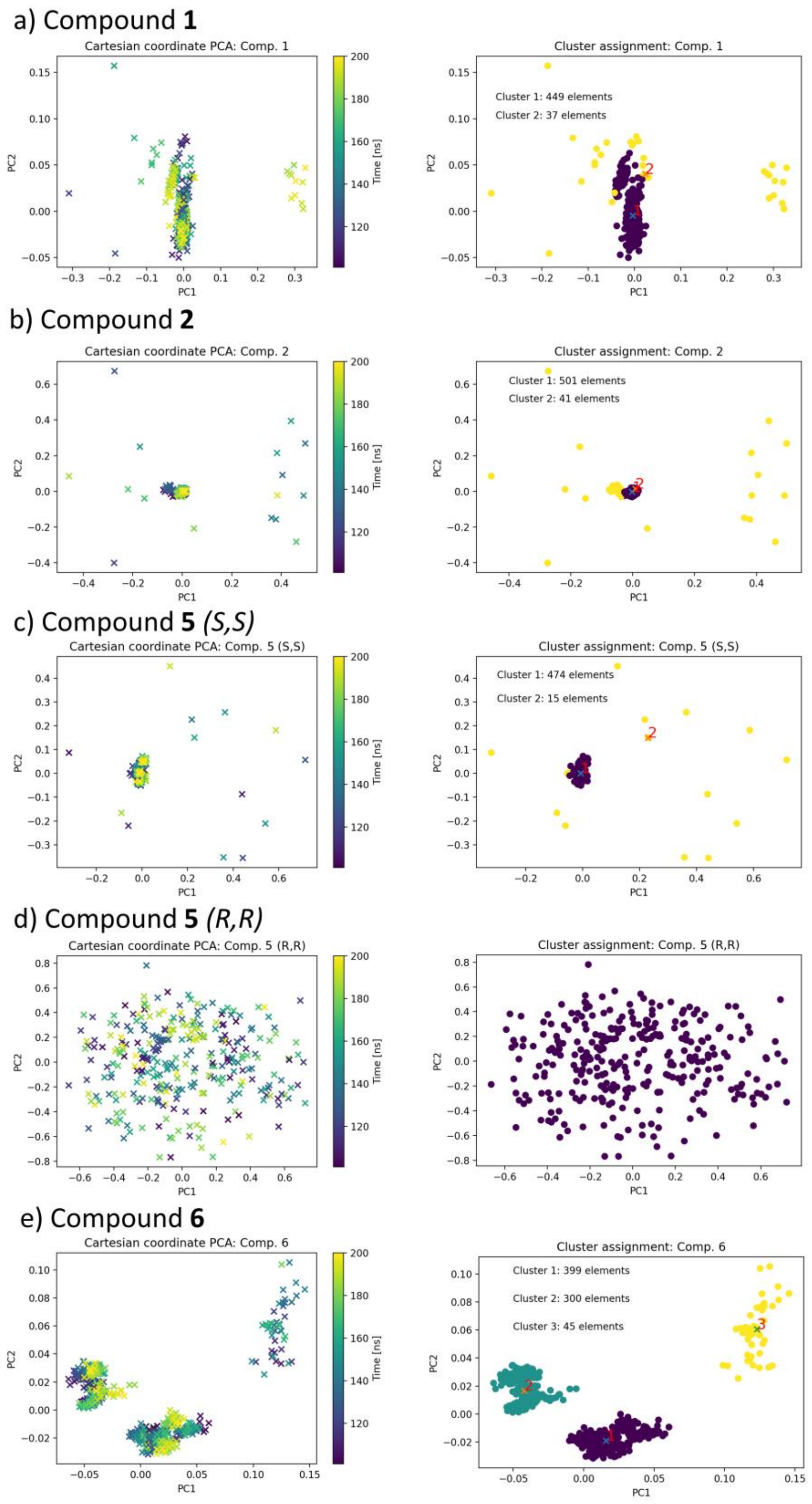

Figure S8. Calculation of representative binding poses for the last 100 ns of each independent simulation. (*Left*) Cartesian coordinates of the heavy atoms for each compound were projected on reduced (principal component) space. Each data point is colored according to time of simulation. (*Right*) Gaussian mixture model cluster assignments per frame (colored by cluster). The centroid point for each cluster (highlighted and numbered in red) was considered as representative pose.

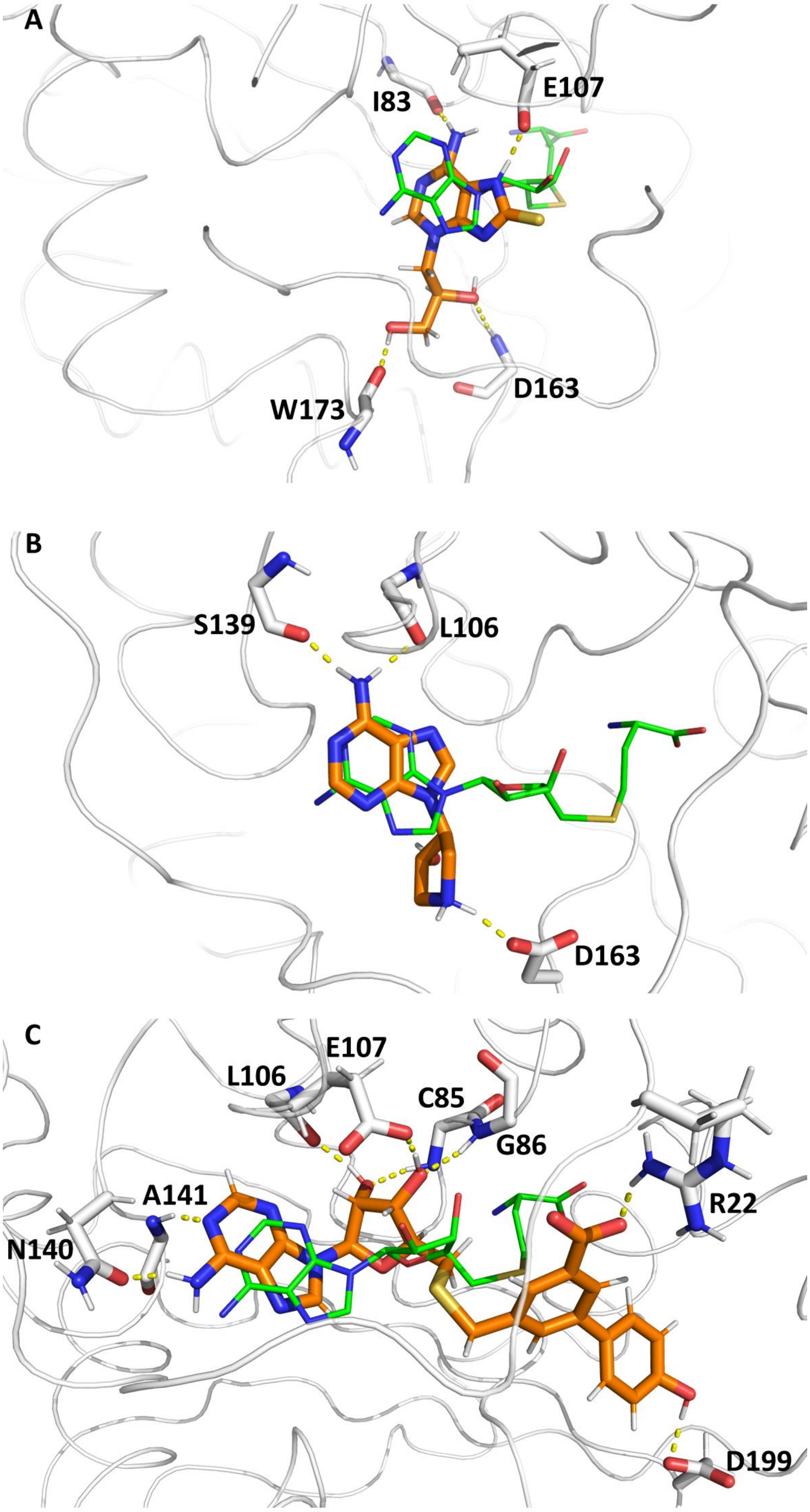

Figure S9. Secondary binding modes of **1** (A), **5**(S,S) (B), and **6** (C) as observed in the MD simulations. The carbon atoms of the binders are in orange and those of the protein in white, hydrogen bonds, and salt bridges are represented as yellow dashed lines. The binding mode of SAH (carbon atoms in green) from the crystal structure of the METTL1-SAH complex (PDB code: 7OGJ) is shown for comparison.

	7OGJ	7PL1
Wavelength	1	1
Resolution range	46.92 - 1.59 (1.647 - 1.59)	40.72 - 1.85 (1.916 - 1.85)
Space group	P 21 21 21	P 43 21 2
Unit cell	63.57 80.46 138.23 90 90 90	128.76 128.76 39.43 90 90 90
Total reflections	781500 (77137)	456918 (41897)
Unique reflections	95912 (9442)	28797 (2783)
Multiplicity	8.1 (8.1)	15.9 (15.0)
Completeness (%)	99.82 (99.40)	99.37 (98.13)
Mean I/sigma(I)	18.42 (1.20)	29.99 (3.13)
Wilson B-factor	24.83	28.46
R-merge	0.0616 (1.509)	0.06876 (0.9389)
R-meas	0.0658 (1.611)	0.07105 (0.972)
R-pim	0.02279 (0.5571)	0.01767 (0.2487)
CC1/2	0.999 (0.608)	1 (0.835)
CC*	1 (0.87)	1 (0.954)
Reflections used in refinement	95783 (9438)	28787 (2783)
Reflections used for R-free	4791 (473)	1441 (139)
R-work	0.1930 (0.3745)	0.1900 (0.2436)
R-free	0.2150 (0.3955)	0.2120 (0.2877)
CC(work)	0.953 (0.625)	0.953 (0.855)
CC(free)	0.937 (0.490)	0.945 (0.755)
Number of non-hydrogen atoms	4092	1897
macromolecules	3425	1647
ligands	86	66
solvent	581	184
Protein residues	425	210
RMS(bonds)	0.007	0.008
RMS(angles)	1.01	0.96
Ramachandran favored (%)	98.8	98.53
Ramachandran allowed (%)	1.2	1.47
Ramachandran outliers (%)	0	0
Rotamer outliers (%)	0.28	0.61
Clashscore	6.24	7.69
Average B-factor	31.22	31.31
macromolecules	29.42	29.99
ligands	42.68	37.26
solvent	40.1	40.99

Table S4. Data collection and refinement statistics for METTL1 in complex with SAH (PDB code: 7OGJ) and METTL1 in complex with sinefungin (PDB code: 7PL1) as generated by phenix.table one. Statistics for the highest-resolution shell are shown in parentheses.

HPLC traces of compounds 6-11

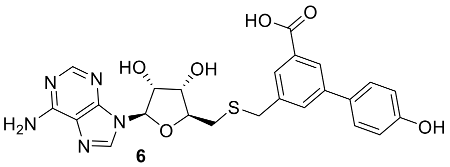

6

Default Individual Report

SAMPLE INFORMATION

Sample Name:	OBV-230	Acquired By:	Olita
Sample Type:	Unknown	Sample Set Name:	Olga_20210518
Vial:	2	Acq. Method Set:	MeCN_0_100%
Injection #:	1	Processing Method:	Processing
Injection Volume:	20.00 ul	Channel Name:	W2489 ChB
Run Time:	20.0 Minutes	Proc. Chnl. Descr.:	W2489 ChB 254nm
Date Acquired:	2021.05.18. 17:38:52 EET		
Date Processed:	2021.05.19. 11:34:00 EET		

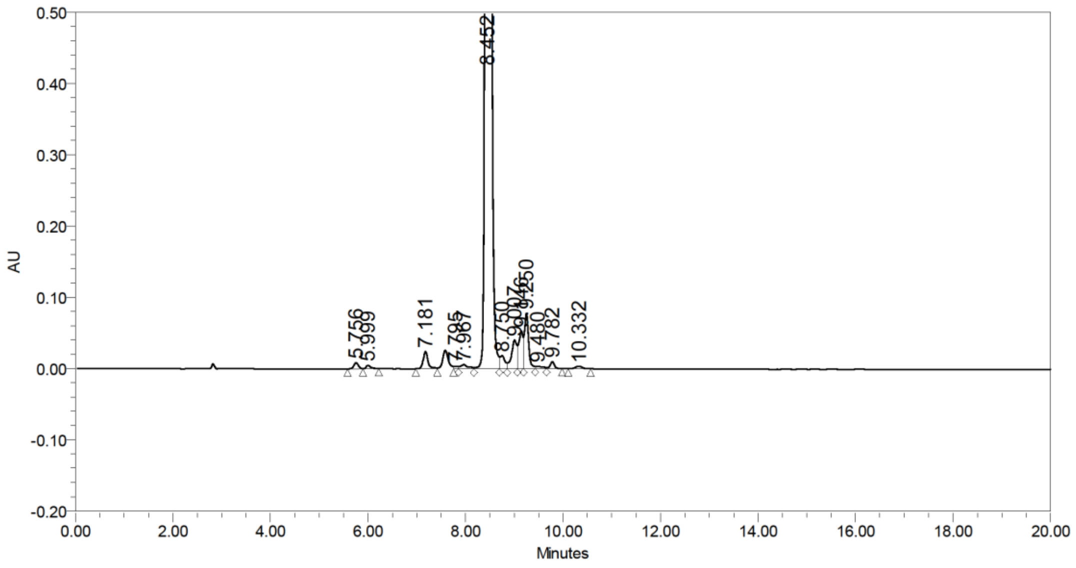

	RT	Area	% Area	Height
1	5.756	55410	0.28	8608
2	5.999	30037	0.15	4638
3	7.181	168506	0.86	24393
4	7.795	20268	0.10	3492
5	7.967	65104	0.33	5825
6	8.452	18009393	91.40	2812469
7	8.750	131797	0.67	18340
8	9.007	306224	1.55	40336

Reported by User: Olita
Report Method: Default Individual Report
Report Method ID 5088
Page: 1 of 2

Project Name: Martins
Date Printed:
2021.05.19.
11:34:51 Europe/Riga

	RT	Area	% Area	Height
9	9.146	330497	1.68	52883
10	9.250	457505	2.32	77597
11	9.480	36460	0.19	3414
12	9.782	59610	0.30	9893
13	10.332	33978	0.17	3275

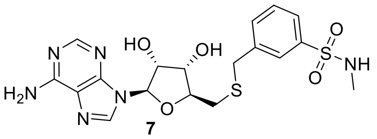

7

Default Individual Report

SAMPLE INFORMATION

Sample Name:	ES-23-4	Acquired By:	Olita
Sample Type:	Unknown	Sample Set Name:	Olga_20210608
Vial:	4	Acq. Method Set:	MeCN_0_100%
Injection #:	1	Processing Method:	Processing
Injection Volume:	20.00 ul	Channel Name:	W2489 ChB
Run Time:	20.0 Minutes	Proc. Chnl. Descr.:	W2489 ChB 254nm

Date Acquired: 2021.06.08. 8:59:00 EET
Date Processed: 2021.06.08. 9:53:31 EET

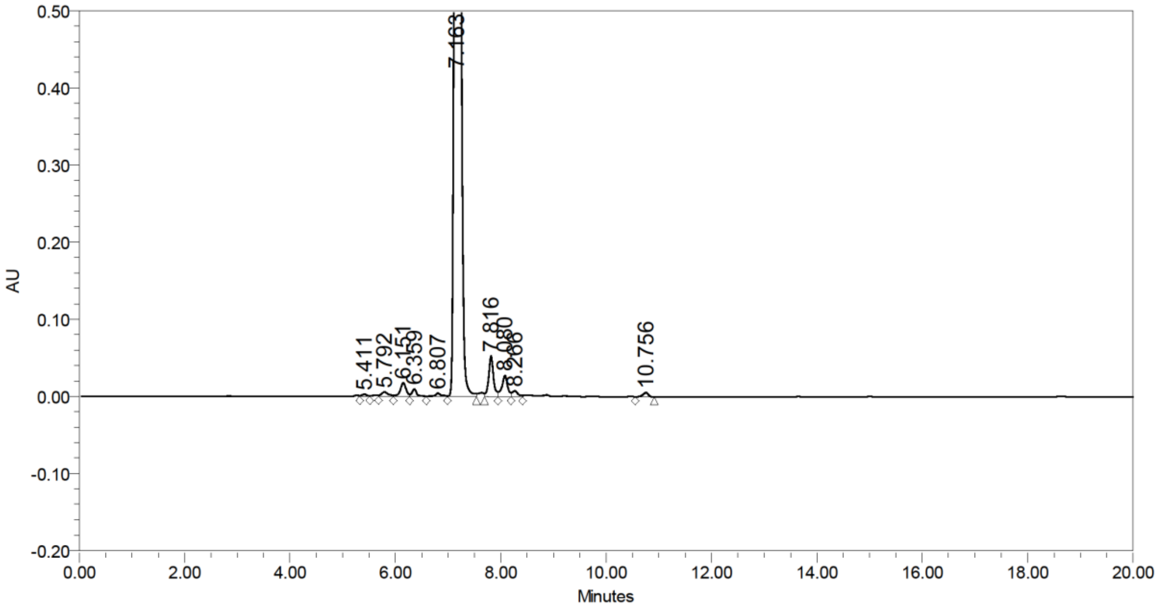

	RT	Area	% Area	Height
1	5.411	22095	0.15	3184
2	5.792	58362	0.39	6043
3	6.151	140283	0.95	17771
4	6.359	66047	0.45	9964
5	6.807	41802	0.28	4618
6	7.163	13847920	93.45	2140438
7	7.816	334560	2.26	52714
8	8.080	207821	1.40	27667

Reported by User: Olita
Report Method: Default Individual Report
Report Method ID 5088
Page: 1 of 2

Project Name: Martins
Date Printed:
2021.06.08.
9:54:25 Europe/Riga

	RT	Area	% Area	Height
9	8.266	59413	0.40	7663
10	10.756	40564	0.27	5616

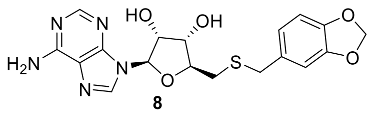

8

Default Individual Report

S A M P L E   I N F O R M A T I O N

Sample Name:	OBV-141	Acquired By:	Olita
Sample Type:	Unknown	Sample Set Name:	
Vial:	5	Acq. Method Set:	MeCN_0_100%
Injection #:	1	Processing Method:	Processing
Injection Volume:	10.00 ul	Channel Name:	W2489 ChB
Run Time:	20.0 Minutes	Proc. Chnl. Descr.:	W2489 ChB 254nm
Date Acquired:	2020.12.23. 11:10:16 EET		
Date Processed:	2020.12.23. 11:51:17 EET		

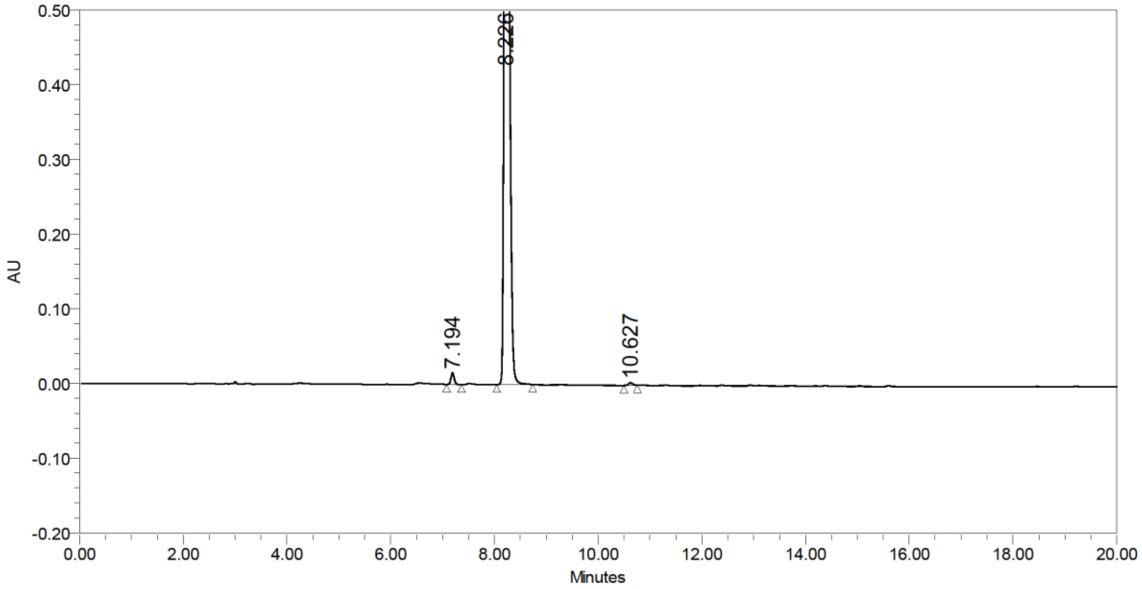

	RT	Area	% Area	Height
1	7.194	67545	0.79	15574
2	8.226	8412372	98.99	1390846
3	10.627	18204	0.21	3379

Reported by User: Olita
Report Method: Default Individual Report
Report Method ID 5088
Page: 1 of 1

Project Name: Martins
Date Printed:
2020.12.23.
11:52:30 Europe/Riga

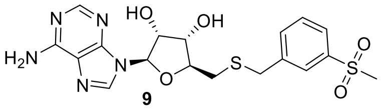

9

Empower2 software

Default Individual Report

SAMPLE INFORMATION

Sample Name:	ES-13-5gat	Acquired By:	Olita
Sample Type:	Unknown	Sample Set Name:	
Vial:	29	Acq. Method Set:	MeCN_0_100%
Injection #:	1	Processing Method:	Processing
Injection Volume:	20.00 ul	Channel Name:	W2489 ChB
Run Time:	20.0 Minutes	Proc. Chnl. Descr.:	W2489 ChB 254nm
Date Acquired:	2021.03.16. 17:01:10 EET		
Date Processed:	2021.03.16. 18:05:51 EET		

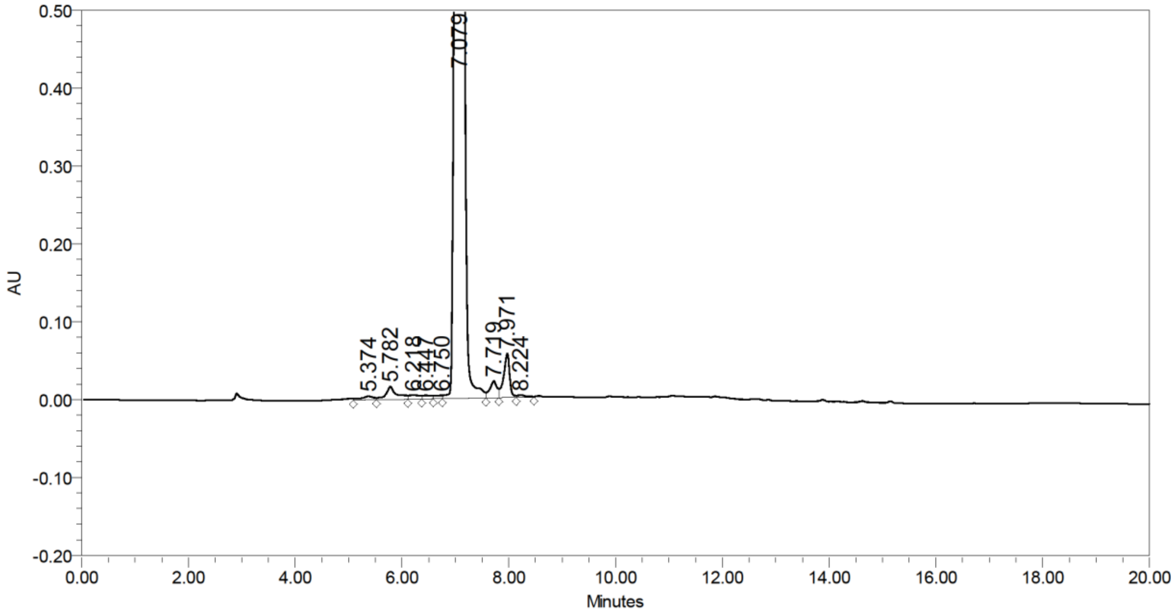

	RT	Area	% Area	Height
1	5.374	76345	0.35	4586
2	5.782	242504	1.10	16618
3	6.218	73734	0.33	5129
4	6.447	55033	0.25	4462
5	6.750	40338	0.18	4161
6	7.079	20882114	94.69	2644567
7	7.719	202748	0.92	21484
8	7.971	442905	2.01	57032

Reported by User: Olita
Report Method: Default Individual Report
Report Method ID 5088
Page: 1 of 2

Project Name: Martins
Date Printed:
2021.03.16.
18:06:27 Europe/Riga

	RT	Area	% Area	Height
9	8.224	36468	0.17	3006

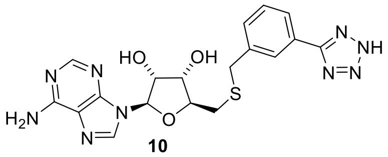

10

Empower2 software

Default Individual Report

SAMPLE INFORMATION

Sample Name:	OBV-218	Acquired By:	Olita
Sample Type:	Unknown	Sample Set Name:	Olga_20210426
Vial:	4	Acq. Method Set:	MeCN_0_100%
Injection #:	1	Processing Method:	Processing
Injection Volume:	20.00 ul	Channel Name:	W2489 ChB
Run Time:	20.0 Minutes	Proc. Chnl. Descr.:	W2489 ChB 254nm
Date Acquired:	2021.04.26. 10:22:18 EET		
Date Processed:	2021.04.26. 11:02:03 EET		

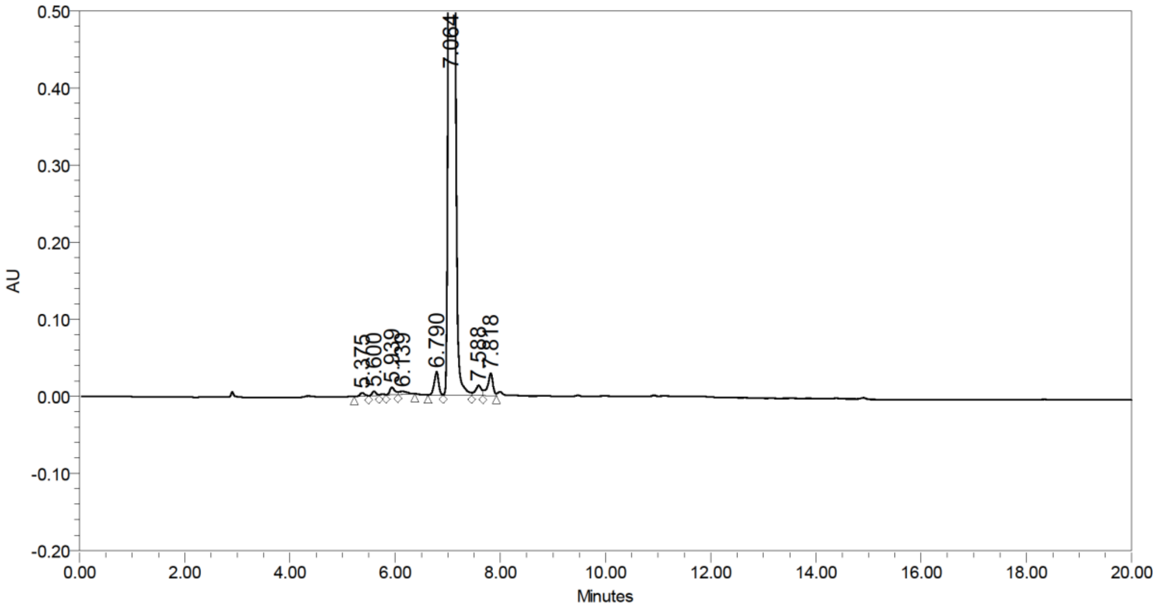

	RT	Area	% Area	Height
1	5.375	28336	0.16	4484
2	5.600	31678	0.17	5848
3	5.939	68638	0.38	9814
4	6.139	41866	0.23	3990
5	6.790	176140	0.97	30233
6	7.064	17539889	96.40	2864629
7	7.588	105890	0.58	13175
8	7.818	202515	1.11	29068

Reported by User: Olita
Report Method: Default Individual Report
Report Method ID 5088
Page: 1 of 1

Project Name: Martins
Date Printed:
2021.04.26.
11:02:43 Europe/Riga

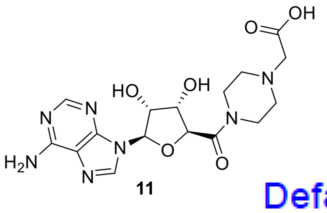

11

Empower software

Default Individual Report

Reported by User: System Project Name: CLI

SAMPLE INFORMATION

Sample Name:	KFSL_OBV-069	Acquired By:	System
Sample Type:	Unknown	Date Acquired:	10/9/2020 12:22:04 PM
Vial:	16	Acq. Method Set:	MeCN_B_0%
Injection #:	1	Date Processed:	10/9/2020 12:44:03 PM
Injection Volume:	10.00 ul	Processing Method:	Certificate_100%_n
Run Time:	20.0 Minutes	Channel Name:	2487Channel 1
Sample Set Name:		Proc. Chnl. Descr.:	

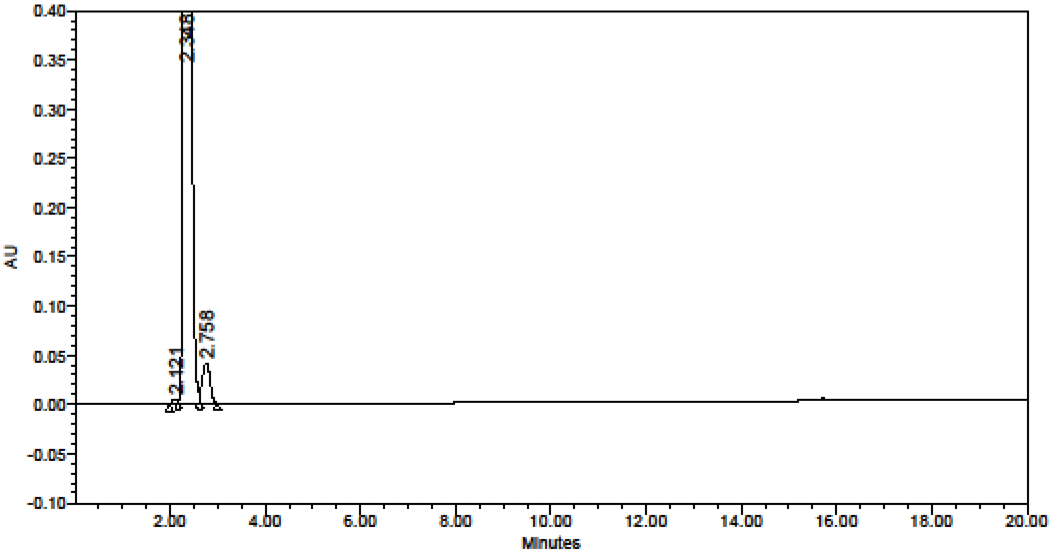

	RT	Area	% Area	Height
1	2.121	33872	0.18	4794
2	2.348	18763282	97.47	2313833
3	2.758	453372	2.36	40926

Report Method: Default Individual Report Printed 12:44:20 PM 10/9/2020 Page: 1 of 1
